# Single‐Nucleus Multi‐Omics Reveals Hypoxia‐Driven Angiogenic Programs and Their Epigenetic Control in Sinonasal Squamous Cell Carcinoma

**DOI:** 10.1002/advs.202510302

**Published:** 2026-01-07

**Authors:** Chaelin You, Jaewoo Park, Jung Yeon Jang, Junho Noh, Jaehyun Lee, Geunho Kwon, Myeong Sang Yu, Yoo‐Sam Chung, Seung‐Jun Lee, Keunsoo Kang, Jihwan Park, Ji Heui Kim, Kyuho Kang

**Affiliations:** ^1^ Department of Biological Sciences and Biotechnology Chungbuk National University Cheongju Republic of Korea; ^2^ Department of Otorhinolaryngology‐Head and Neck Surgery Asan Medical Center University of Ulsan College of Medicine Seoul Republic of Korea; ^3^ Research Institute of Pharmaceutical Sciences and College of Pharmacy Seoul National University Seoul Republic of Korea; ^4^ Department of Microbiology College of Science & Technology Dankook University Cheonan Republic of Korea; ^5^ School of Life Sciences Gwangju Institute of Science and Technology (GIST) Gwangju Republic of Korea

**Keywords:** hypoxia‐angiogenesis axis, multi‐omic profiling, rare cancer genomics, sinonasal squamous cell carcinoma, tumor heterogeneity

## Abstract

Sinonasal squamous cell carcinoma (SNSCC) is a rare malignancy with poorly understood molecular drivers. Consequently, its cellular composition and tumor microenvironment (TME) remain largely undefined. Here, we performed integrated bulk and single‐nucleus multi‐omic analyses to map the SNSCC ecosystem. Within the malignant compartment, we identified five distinct populations, with hypoxic (TC1) and proliferative (TC2) subtypes associated with adverse clinical outcomes. Functionally, TC1 cells orchestrate a hypoxia‐driven angiogenic program via coordinated secretion of adrenomedullin (ADM), MIF, and VEGFA, promoting endothelial tip cell (EC1) differentiation. Integrative analysis revealed these transcriptional programs are underpinned by tumor‐specific chromatin accessibility and DNA hypomethylation, particularly at AP‐1‐enriched regulatory elements. Mechanistically, in vitro studies confirmed that this response depends on cooperative AP‐1 and HIF1A signaling. Furthermore, histological analysis of patient tissues demonstrated spatial co‐localization of GLUT1‐expressing TC1 cells with DLL4‐positive EC1 cells. These findings elucidate the epigenetic landscape underlying tumor–stromal interactions and establish the ADM/VEGFA axis as a critical therapeutic target to disrupt epigenetically controlled angiogenesis in SNSCC.

## Graphical abstract

Single‐nucleus multi‐omics profiling of sinonasal squamous cell carcinoma unveils a hypoxia‐driven angiogenic axis. A specific hypoxic tumor subpopulation orchestrates endothelial tip cell differentiation via epigenetically regulated ADM and VEGFA secretion. This study elucidates the AP‐1/HIF1A‐mediated regulatory landscape underlying tumor–stromal interactions, establishing a mechanistic basis for targeted therapeutic intervention in this aggressive malignancy.

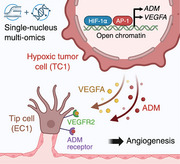

## 1 Introduction

Sinonasal squamous cell carcinoma (SNSCC) represents a distinct subset of head and neck malignancies, accounting for less than 1% of all malignant neoplasms and 5% of head and neck cancers [1, 2, 3]. As the predominant histological subtype of sinonasal cancer, SNSCC exhibits unique clinicopathological features that set it apart from other head and neck malignancies [4, 5]. Despite therapeutic advances, the 5‐year survival rate remains between 30%–50%, showing minimal improvement over recent decades [1, 6, 7, 8]. The rarity of SNSCC creates substantial challenges for research and treatment, including limited specimen availability and diagnostic complexity [9, 10]. Current treatment protocols are largely adapted from those designed for more common malignancies, emphasizing the need for targeted research into SNSCC‐specific disease mechanisms.

Recent technological advancements in transcriptomics and epigenomics have significantly enhanced our understanding of cancer biology, including rare malignancies [11]. Bulk RNA sequencing (RNA‐seq) has revealed gene expression patterns across complex tumor tissues [12], while comprehensive epigenetic profiling has illuminated regulatory mechanisms governing oncogenic gene transcription [13, 14]. Within SNSCC research, transcriptome analyses have yielded potential biomarkers and characterized distinct molecular subtypes [15, 16, 17, 18]. Large‐scale DNA methylation profiling has further refined the classification of sinonasal cancers, especially undifferentiated carcinomas, underscoring the role of epigenetic regulation [19].

The tumor microenvironment (TME) significantly influences cancer progression and treatment outcomes [20, 21]. Single‐cell technologies, including RNA‐seq and epigenomic profiling, have revealed cellular heterogeneity and diverse phenotypes within tumors [22, 23]. Multi‐omic approaches that combine gene expression and chromatin accessibility analyses at the single‐cell level have provided detailed insights into cellular identity and properties across various cancers [24, 25, 26]. These approaches have proven particularly valuable in a rare cancer, as demonstrated by recent work in desmoplastic small round cell tumor that revealed heterogeneous features of tumor landscapes and clinically relevant gene signatures [27]. Despite these technological advances, the cellular composition and molecular networks within the SNSCC microenvironment remain undefined, highlighting the need for comprehensive single‐cell multi‐omic analysis.

Hypoxia emerges as a critical feature of the SNSCC microenvironment requiring further investigation. This hallmark feature of solid tumors promotes aggressive phenotypes and contributes to treatment resistance [28, 29]. Clinical studies have demonstrated substantial overexpression of hypoxia‐associated proteins in SNSCC patients, with CA‐IX and VEGF present in 85% of cases, VEGFR1 in 75% of cases, and GLUT1 in 62.5% of cases [30]. The presence of heterogeneous necrotic regions in larger tumors provides additional evidence of chronic hypoxic conditions [31, 32]. A deeper understanding of how hypoxia influences transcriptional regulation, intercellular communication, and critical processes such as angiogenesis in SNSCC could illuminate new therapeutic strategies.

Our integrated analysis of bulk and single‐nucleus multi‐omic data from tumor and normal nasal mucosa provides a comprehensive molecular characterization of SNSCC. This approach identified five distinct malignant cell populations, with hypoxic (TC1) and proliferative (TC2) subsets showing significant association with poor clinical outcomes. We found that tumor‐specific changes in chromatin accessibility and DNA methylation, particularly at *cis*‐regulatory elements with AP‐1 binding motifs, underpin the transcriptional signatures driving malignant phenotypes. Crucially, by combining multi‐omic profiling with functional in vitro studies, we delineate a hypoxia‐driven angiogenic axis orchestrated by TC1 cells through the coordinated secretion of adrenomedullin (ADM), MIF, and VEGFA, promoting endothelial tip cell (EC1) differentiation. Direct histological evidence from patient tumor tissues, demonstrating spatial co‐localization of GLUT1 and DLL4, supports this physical link between the hypoxic compartment and the angiogenic response. Our findings thus elucidate the epigenetic mechanisms and critical tumor–stromal interactions within the SNSCC ecosystem, highlighting the coordinated regulation by AP‐1 and HIF1A. These results establish the ADM/VEGFA axis as a key molecular determinant of progression and a critical targetable pathway to disrupt the epigenetically controlled angiogenesis in SNSCC.

## 2 Results

### 2.1 Molecular Profiling Reveals Distinct Transcriptional Programs in SNSCC

**FIGURE 1:**
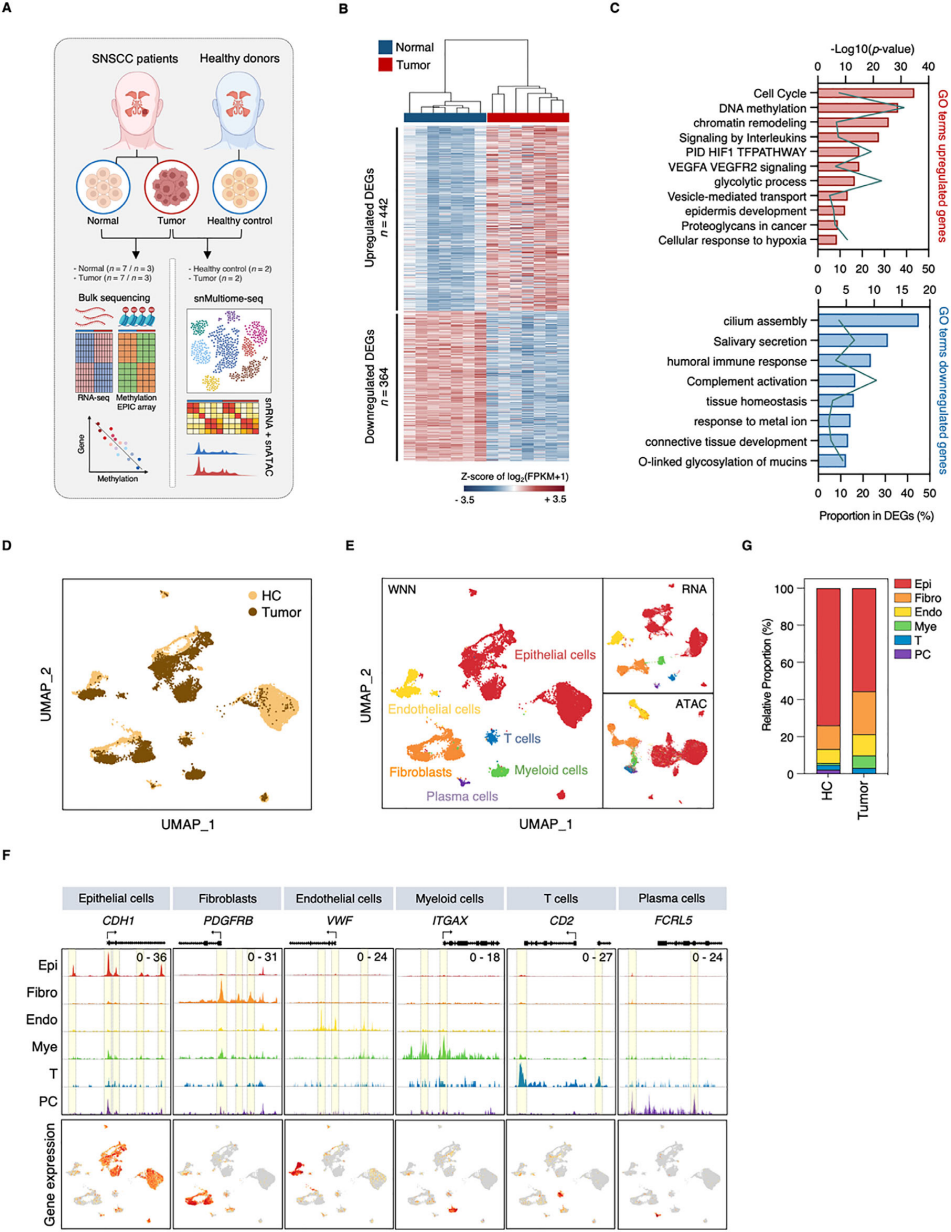
Integrated transcriptomic analysis reveals cellular heterogeneity and molecular signatures of SNSCC. (A) Experimental design and multi‐omic profiling strategy for analyzing sinonasal squamous cell carcinoma (SNSCC) samples (Created in BioRender. BioRender.com/arddqn0). (B) Differential gene expression analysis between tumor and normal tissues identifies two distinct clusters through K‐means analysis (k = 2), with genes selected based on |log2 fold change| > 1, adjusted *p* < 0.05, and fragments per kilobase of transcript per million mapped reads (FPKM) > 2. Data are shown as Z‐transformed log2(FPKM+1) values. (C) Gene ontology (GO) enrichment analysis of differentially expressed genes (DEGs) between tumor and normal tissues was performed using METASCAPE. (D) Uniform manifold approximation and projection (UMAP) dimensionality reduction of 17,648 nuclei isolated from SNSCC (*n* = 2) and healthy control (HC, *n* = 2) tissues, with points colored by tissue origin. (E) Cell type identification through weighted nearest neighbor (WNN) analysis integrating RNA and ATAC profiles, with UMAP projections showing clustering by WNN, RNA expression, and chromatin accessibility. (F) Differential chromatin accessibility profiles (Upper) and corresponding gene expression patterns (Lower) of cell type‐specific marker genes across identified populations, with cell type‐specific peaks highlighted. (G) Quantitative analysis of major cell type distributions in SNSCC compared to healthy control (HC) tissues.

To establish a comprehensive molecular profile of SNSCC, we performed integrated transcriptomic and epigenomic analyses of tumor and normal tissues from SNSCC patients, along with nasal mucosa from healthy donors, using both bulk and single‐nucleus approaches (Figure 1A, Tables 1 and 2). Bulk RNA‐seq of surgical specimens from eight SNSCC patients (seven tumor and seven normal tissues) revealed clear transcriptional differences between tumor and normal tissues. Principal component analysis (PCA) demonstrated robust separation between tumor and normal samples (Figure S1A), with differential expression analysis identifying 806 significantly altered genes (442 upregulated, 364 downregulated; adjusted *p*‐value < 0.05, |log2 fold change| > 1) (Figure 1B).

**TABLE 1:** Summary of sample information for multi‐omic profiling.

Sample ID	Tissue type	Paired normal tissue	Bulk RNA‐seq	DNA methylation EPIC array	snRNA/ATAC‐seq
CA1	Tumor	N2	Y	Y	—
CA2	Tumor	N3	Y	—	—
CA5	Tumor	N6	Y	Y	Y
CA6	Tumor	N7	Y	—	—
CA7	Tumor	—	Y	—	—
CA8	Tumor	N9	Y	Y	Y
CA9	Tumor	N10	Y	—	—
N2	Normal	—	Y	Y	—
N3	Normal	—	Y	Y	—
N6	Normal	—	Y	Y	—
N7	Normal	—	Y	—	—
N9	Normal	—	Y	—	—
N10	Normal	—	Y	—	—
N11	Normal	—	Y	—	—
HC1	Healthy control	—	—	—	Y
HC2	Healthy control	—	—	—	Y

**TABLE 2:** Clinical data for patients with sinonasal squamous cell carcinoma.

Patient ID	Sex	Age	Subsite	Primary	T	Histology	IP^d^_Inverted	Tumor grade	Lymphovascular invasion	Perineural invasion
CA1	M	71	FS^a^	Primary	4b	SCC	IP background	3	Neg	Pos
CA2	M	48	MS^b^	Primary	4a	SCC	—	2	N/A	Pos
CA5	M	75	NC^c^	Primary	4a	SCC	—	2	Pos	Neg
CA6	M	73	FS	Rec	4b	SCC	—	2	Neg	Neg
CA7	F	70	FS	Rec	4b	SCC	IP background	1	N/A	N/A
CA8	M	61	NC	Primary	4a	SCC	IP background	2	Pos	Neg
CA9	M	78	MS	Primary	4a	SCC	—	2	Neg	Neg

^a^ Frontal sinus;

^b^ Maxillary sinus;

^c^ Nasal cavity;

^d^ Inverted papilloma.

Gene Ontology (GO) analysis revealed distinct biological programs characterizing SNSCC (Figure 1C). Upregulated genes showed significant enrichment in cell cycle‐related processes, with elevated expression of key regulators including *MKI67* and *TOP2A* (Figure 1C; Figure S1B). Notably, genes involved in epigenetic regulation, particularly DNA methylation and chromatin remodeling, were markedly enriched, including critical modulators such as the DNA methyltransferase *DNMT1* and histone demethylase *KDM3A* (Figure 1C; Figure S1B). The analysis also revealed significant metabolic reprogramming, with enrichment of HIF1 transcription factor (TF) signaling, glycolytic processes, and hypoxic response pathways, exemplified by increased expression of *HIF1A* and the glucose transporter *SLC2A1* (Figure 1C; Figure S1B). Gene Set Enrichment Analysis (GSEA) corroborated these findings, demonstrating significant enrichment of E2F targets, G2M checkpoint genes, and hypoxia‐related pathways among upregulated genes (Figure S1C). Conversely, downregulated genes primarily were associated with cilium assembly, salivary secretion, and mucin O‐glycosylation, reflecting compromised mucosal epithelial function (Figure 1C). Specifically, expression of mucin genes essential for airway epithelial defense (*MUC5AC* and *MUC5B*) showed a significant reduction in tumor tissues (Figure S1B).

The transcriptional landscape further revealed distinctive immunological features. Upregulated genes showed enrichment in interleukin signaling pathways, while downregulated genes were associated with humoral immunity and complement activation (Figure 1C). This pattern manifested through elevated expression of immune regulatory factors such as *CCL3* and *SPP1*, concurrent with reduced expression of humoral response mediators (*DMBT1* and *XBP1*) and complement components (*C3* and *C4A*) (Figure S1B). These results establish that SNSCC exhibits a characteristic molecular profile defined by dysregulated cell cycle control, altered epigenetic programming, metabolic adaptation, and immune modulation that fundamentally distinguishes malignant from normal tissue.

### 2.2 Single‐Nucleus Multi‐Omic Analysis Reveals Complex Cellular Architecture of SNSCC

To elucidate the TME of SNSCC at single‐cell resolution, we performed integrated single‐nucleus RNA and ATAC sequencing (snRNA/ATAC‐seq) analysis of tumor tissues from SNSCC patients (*n* = 2) and healthy nasal mucosa (*n* = 2; designated as healthy control, HC) using the 10× Genomics Multiome platform, enabling concurrent profiling of gene expression and chromatin accessibility (Figure 1A). Following quality control and batch effect correction using Harmony, we analyzed 18 319 nuclei. Integration of RNA and ATAC profiles through weighted nearest neighbor (WNN) analysis revealed 28 distinct clusters representing six major cell types (Figure 1D,E; Figure S1D,E). We excluded ambiguous clusters showing either multiple lineage markers or poor quality metrics from subsequent analyses (Figure S1E,F). Cell type identification was supported by both transcriptional and chromatin accessibility signatures of established lineage markers (Figure 1F; Figure S1E), including epithelial cell (*CDH1*, *EPCAM*, and *KRT8*), fibroblast (*DCN*, *PDGFRB*, and *THY1*), endothelial cell (*CD34*, *CDH5*, and *VWF*), myeloid cell (*ITGAX*, *MS4A6A*, and *SPI1*), T cell (*CD2*, *CD3D*, and *CD3G*), and plasma cell (*FCRL5*, *JCHAIN*, and *MZB1*) populations.

Quantitative analysis of cellular composition revealed epithelial cells as the predominant population in both tumor and healthy tissues (Figure 1G). Notably, SNSCC demonstrated significant alterations in immune cell distribution, with increased myeloid cell and decreased plasma cell proportions compared to HCs (Figure 1G; Figure S1G). To validate these findings in a larger cohort, we performed deconvolution analysis of bulk RNA‐seq data using CIBERSORTx to estimate the abundance of 22 immune cell populations. This analysis corroborated the single‐cell findings, confirming significant expansion of macrophage populations and reduction in plasma cells in SNSCC (Figure S1H,I). These cellular composition changes were further reflected in the differential expression of lineage‐specific markers, including elevated expression of the macrophage marker *CD68* and reduced expression of the plasma cell marker *JCHAIN* (Figure S1J).

### 2.3 Cell Type‐Specific Transcriptional Programs Reveal Distinct Oncogenic Signatures

**FIGURE 2:**
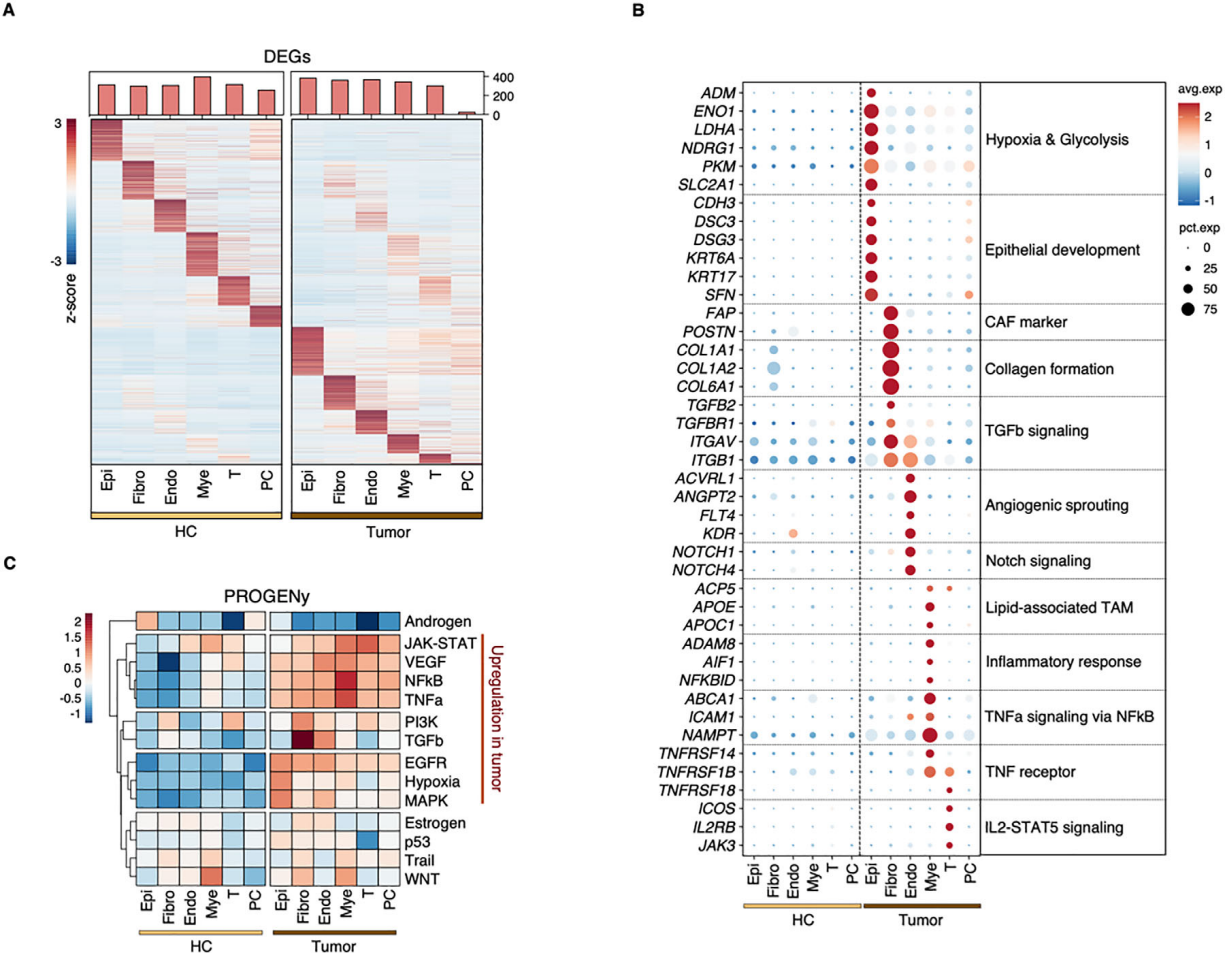
Single‐cell transcriptome analysis reveals distinct cellular states and pathway activation in SNSCC. (A) Differential gene expression analysis across cell types identified in SNSCC and HC tissues, displayed as a heatmap. (B) Cell type‐specific functional characteristics and associated gene expression patterns in SNSCC, represented as a dot plot with size indicating the percentage of expressing cells and color intensity reflecting expression magnitude. (C) PROGENy pathway activity scores across 14 signaling pathways, comparing cell type‐specific activation states between SNSCC and HC tissues.

Differential expression analysis across cell types revealed distinct transcriptional programs between tumor and HC environments (adjusted *p*‐value < 0.05, log2 fold change > 1) (Figure 2A). Plasma cells were excluded from detailed analysis due to their limited representation (<20 cells) in tumor tissues. Functional characterization of tumor‐specific populations unveiled key pathways associated with SNSCC progression (Figure 2B,C; Figure S2A). Malignant epithelial cells demonstrated significant upregulation of hypoxia‐ and glycolysis‐associated genes (*ADM*, *ENO1*, *LDHA*, *NDRG1*, *PKM*, and *SLC2A1*) alongside epithelial development markers (*CDH3*, *DSC3*, *DSG3*, *KRT6A*, *KRT17*, and *SFN*), corroborating our bulk sequencing findings (Figure 2B). GO pathways in healthy nasal mucosa reflected cell type‐specific characteristics (Figure S2B). HC epithelial cells were enriched for salivary secretion‐related genes (*CCL28*, *DMBT1*, *EGF*, and *STATH*), consistent with our bulk RNA‐seq findings and reflecting the airway's ciliated and secretory cell composition (Figure S2B,C).

Tumor‐associated fibroblasts exhibited characteristic expression of established cancer‐associated fibroblast (CAF) markers (*FAP* and *POSTN*), coupled with elevated expression of collagen formation genes (*COL1A1*, *COL1A2*, and *COL6A1*) and TGFβ signaling components (*TGFB2*, *TGFBR1*, *ITGAV*, and *ITGB1*) (Figure 2B). In contrast, normal fibroblasts showed higher expression of muscle structure‐related genes (*ACTA2*, *FHL1*, *MYH11*, and *MYOCD*) compared to CAFs (Figure S2B,C). Endothelial cells from tumor tissue showed significant enrichment in angiogenesis‐related processes, particularly VEGFA‐VEGFR2 signaling and vascular sprouting (Figure S2A). Notable upregulation of genes associated with angiogenic sprouting (*ACVRL1*, *ANGPT2*, *FLT4*, and *KDR*) and NOTCH signaling (*NOTCH1* and *NOTCH4*) suggested active angiogenesis in SNSCC (Figure 2B).

The predominant immune population in SNSCC, myeloid cells (Figure 1G), displayed complex phenotypes characterized by lipid‐associated tumor‐associated macrophage (TAM) signatures (*ACP5*, *APOE*, and *APOC1*) and inflammatory response genes (*ADAM8*, *AIF1*, and *NFKBID*). TNF‐NFκB signaling components (*ABCA1*, *ICAM1*, and *NAMPT*) showed significant upregulation in tumor‐associated myeloid cells (Figure 2B). Tumor‐infiltrating T cells exhibited enrichment in primary immunodeficiency and JAK‐STAT signaling pathways, with elevated expression of TNF receptors (*TNFRSF14*, *TNFRSF1B*, and *TNFRSF18*) and IL2‐STAT5 signaling genes (*ICOS*, *IL2RB*, and *JAK3*) (Figure 2B; Figure S2A).

PROGENy pathway analysis revealed elevated activity of multiple oncogenic pathways in tumor tissue, including JAK‐STAT, VEGF, NFκB, TNF, PI3K, TGFβ, EGFR, Hypoxia, and MAPK signaling (Figure 2C). Notably, EGFR, Hypoxia, and MAPK pathways showed particular enhancement in tumor epithelial cells, while PI3K and TGFβ signaling predominated in tumor fibroblasts. Inflammatory pathways, specifically TNF and NFκB signaling, demonstrated marked activation in tumor‐associated myeloid cells (Figure 2C). Feature plots illustrated the expression patterns of representative upregulated DEGs across tumor and HC tissues (Figure S2D). These findings provide comprehensive insight into the cell type‐specific molecular landscapes that characterize SNSCC pathogenesis.

### 2.4 Genome‐Wide Chromatin Accessibility Analysis Reveals Cell Type‐Specific Regulatory Mechanisms

**FIGURE 3:**
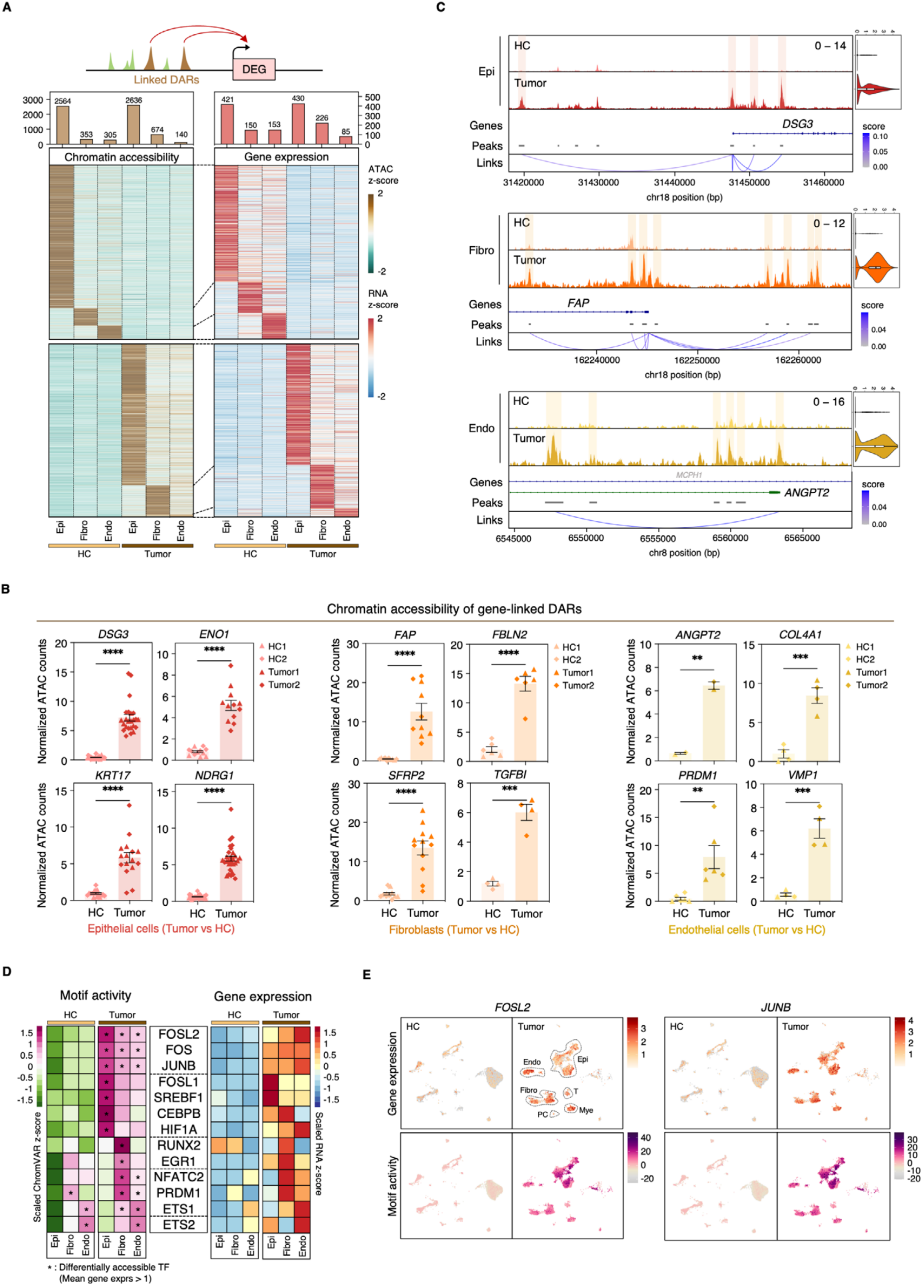
Integrated epigenetic and transcriptional analysis reveals distinct regulatory landscapes in SNSCC. (A) Integration strategy for correlating differentially accessible regions (DARs) with differentially expressed genes (DEGs) (Upper). Paired heatmaps of chromatin accessibility and corresponding gene expression patterns for DAR‐linked DEGs in epithelial cell, fibroblast, and endothelial cell populations, comparing SNSCC to HC samples (Lower). (B) Quantification of chromatin accessibility at cell type‐specific DARs linked to selected DEGs, comparing SNSCC tumors with HCs. Data represent mean ± SEM; statistical significance determined by two‐tailed unpaired *t*‐test (*p < 0.05, **p < 0.01, ***p < 0.001, ****p < 0.0001). (C) Representative genome browser tracks showing cell type‐specific ATAC‐seq signal intensity and corresponding gene expression levels for selected DEGs identified in (B). SNSCC samples are shown in dark colors, while HC samples are shown in light colors; peak coordinates and peak‐gene associations are indicated. (D) Integrated analysis of chromVAR motif activity scores (Left) and corresponding transcription factor (TF) expression levels (Right) for differentially enriched binding motifs showing elevated expression in SNSCC compared to matched normal cell populations. (E) UMAP visualization of *FOSL2* and *JUNB* expression patterns (Upper) and corresponding motif activity scores (Lower), stratified by tissue type.

To characterize the regulatory architecture of SNSCC, we performed a comprehensive analysis of chromatin accessibility patterns across distinct cell populations in tumor and HC tissues. Using stringent criteria (adjusted *p*‐value < 0.05, |log2 fold change| > 1), we identified differentially accessible regions (DARs) specific to each cell type. Given the limited number of DARs (<50) in immune populations, we focused our analysis on the three predominant cell types: epithelial cells, fibroblasts, and endothelial cells (Figure S3A). We utilized the LinkPeaks function to quantify the statistically significant correlation between chromatin accessibility and nearby gene expression. Integration of chromatin accessibility with transcriptional changes revealed a significant correlation between DARs and differentially expressed genes (DEGs) across cell types (Figure 3A), with approximately 10% of DARs showing direct association with DEGs (Figure S3B).

Genomic distribution analysis revealed that DEG‐linked DARs predominantly localized to promoter‐transcription start site (TSS) regions, introns, and intergenic regions, suggesting that *cis*‐regulatory elements play central roles in chromatin remodeling during SNSCC progression (Figure S3C). The remaining DARs were distributed across transcription termination sites, UTRs, exons, and noncoding regions (Figure S3C). Functional analysis of cell type‐specific accessible regions associated with upregulated DEGs corroborated our previous molecular characterization of SNSCC (Figure S3D).

Tumor‐specific cell populations demonstrated distinctive patterns of chromatin accessibility at key regulatory loci. Malignant epithelial cells showed enhanced accessibility at regions controlling *DSG3*, *ENO1*, *KRT17*, and *NDRG1* expression. CAFs exhibited increased accessibility near *FAP*, *FBLN2*, *SFRP2*, and *TGFBI*, while tumor endothelial cells displayed elevated accessibility at regulatory regions of *ANGPT2*, *COL4A1*, *PRDM1*, and *VMP1* (Figure 3B). These accessibility changes strongly correlated with increased expression of corresponding genes (Figure S3E). Cell type‐specific analysis of representative DARs and associated gene expression patterns highlighted distinct functional roles within the TME (Figure 3C). Notably, these included genes previously implicated in cancer progression: DSG3 in head and neck cancer development [33], FAP‐expressing fibroblasts in immunosuppression and therapy resistance [34, 35, 36], and ANGPT2 in tumor angiogenesis [37]. These findings suggest that cell type‐specific alterations in chromatin accessibility drive the expression of key oncogenic programs in SNSCC.

### 2.5 Cell Type‐Specific Transcription Factor Activity Defines Regulatory Programs in SNSCC

Analysis of differential TF binding motif enrichment in tumor and HC tissues (adjusted *p*‐value < 0.05, log2 fold change > 0.58) revealed distinct regulatory programs across cell populations (Figure S3F). Each major cell type exhibited characteristic motif enrichment patterns, exemplified by strong PU.1 (SPI1) enrichment in myeloid cells and IRF4 enrichment in plasma cells (Figure S3F).

We focused our analysis on TFs showing both increased expression in tumor tissue and enriched motif accessibility among moderately expressed factors (mean expression > 1). Integration of ChromVAR motif accessibility scores with gene expression data revealed cell type‐specific regulatory programs (Figure 3D). Malignant epithelial cells showed significant enrichment of HIF1A binding motifs, consistent with the observed hypoxic transcriptional signature. CAFs demonstrated enrichment for RUNX2, EGR1, and NFATC2 motifs, with RUNX2 previously implicated in defining tumorigenic fibroblast populations. Tumor endothelial cells showed particular enrichment for ETS family TFs, notably ETS1 and ETS2, consistent with their established roles in angiogenic regulation.

Notably, AP‐1 family motifs, including FOS, FOSL2, and JUNB, showed broad enrichment across tumor cell populations, with particular prominence in malignant epithelial cells (Figure 3D,E). The expression of *FOSL2* and *JUNB* was significantly elevated in the tumor compared to normal tissue (Figure S3G). The correlation between enhanced expression and motif activity of these factors in tumor cell populations (Figure 3E) suggests a central role for AP‐1‐mediated transcriptional regulation in SNSCC pathogenesis. These findings demonstrate that integrated analysis of chromatin accessibility and TF activity reveals cell type‐specific regulatory mechanisms underlying the complex transcriptional programs driving SNSCC development.

### 2.6 DNA Methylation Profiling Reveals Extensive Epigenetic Reprogramming in SNSCC

Given the enrichment of DNA methylation‐related processes identified in our transcriptional analysis, we performed comprehensive methylation profiling of SNSCC using the EPIC array platform. We analyzed tumor (*n* = 3) and normal tissue (*n* = 3) from patients included in our RNA‐seq cohort, integrating these data with published methylation profiles of 35 SNSCC and 8 normal sinonasal tissues (GSE189778). Multi‐dimensional scaling (MDS) analysis of the 1000 most variable CpG sites demonstrated robust separation between normal and tumor samples, with our methylation profiles showing concordance with published datasets (Figure S4A).

**FIGURE 4:**
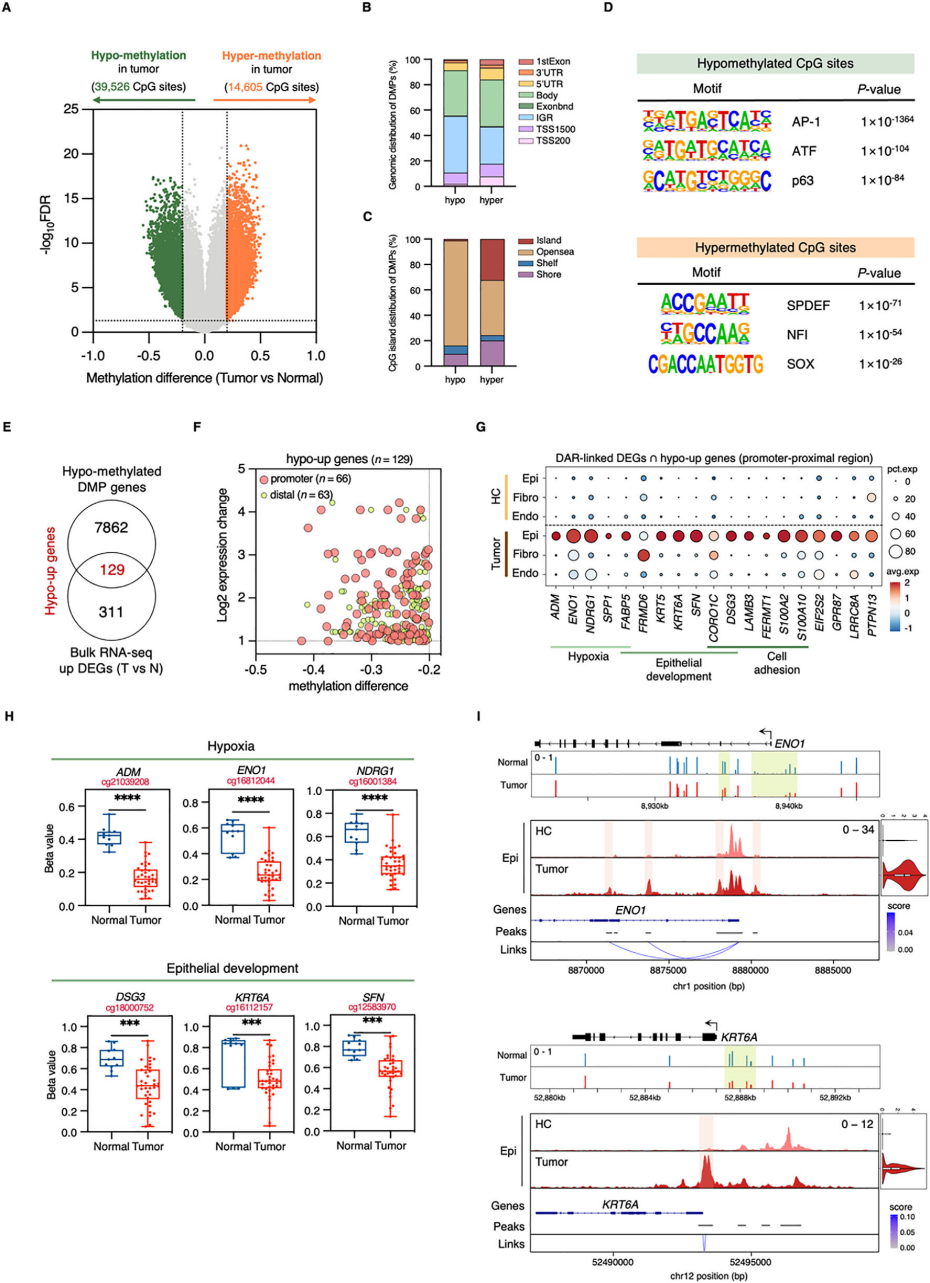
Analysis of DNA methylation patterns reveals extensive epigenetic reprogramming in SNSCC. (A) Genome‐wide differential methylation analysis between SNSCC tumors and normal tissues. Significantly differentially methylated positions (DMPs) defined by |Δβ| > 0.2 and adjusted *p* < 0.05. Nonsignificant CpG sites are shown in gray. (B) Genomic annotation of hypo‐ and hypermethylated DMPs across defined genomic features. (C) Distribution analysis of CpG island (CGI) association for hypo‐ and hypermethylated DMPs. (D) TF binding motifs significantly enriched in hypomethylated (Upper) and hypermethylated (Lower) DMPs identified through *de novo* motif analysis. (E) Overlap analysis identifying hypomethylated genes showing concurrent transcriptional upregulation (hypo‐up) in SNSCC compared to normal tissue. (F) Correlation between DNA methylation changes (Δβ) and gene expression differences (log2 fold change) for 129 hypo‐up genes, stratified by genomic context (promoter, red; distal, green). (G) Expression analysis of DAR‐linked DEGs from Figure 3A intersecting with 66 promoter‐proximal hypo‐up genes across cell types in SNSCC and HC samples. Associated functional annotations are shown below. (H) DNA methylation levels (β‐values) for selected hypo‐up genes identified in (G), comparing SNSCC to normal samples. Box plots show 25th to 75th percentiles with whiskers extending to the minimum and maximum values. Center lines indicate medians. Statistical significance determined by two‐tailed unpaired *t*‐test (*p < 0.05, **p < 0.01, ***p < 0.001, ****p < 0.0001). (I) Integrated visualization of DNA methylation patterns at *ENO1* and *KRT6A* loci in SNSCC (red) and normal tissues (blue) (Upper), with corresponding ATAC‐seq signal intensity and gene expression profiles in epithelial cells (Lower); peak coordinates and peak‐gene associations are indicated.

Differential methylation analysis identified 54,131 significantly altered CpG sites in tumor tissue (39,526 hypomethylated, 14,605 hypermethylated; adjusted *p*‐value < 0.05, |Δβ| > 0.2) (Figure 4A; Figure S4B). Genomic distribution analysis revealed distinct patterns of differential methylation: hypomethylated CpGs showed enrichment in intergenic regions, while hypermethylated CpGs predominantly localized near TSSs, including TSS200, TSS1500, 5'UTR, and first exon regions (Figure 4B). Analysis of CpG island (CGI) distribution demonstrated preferential hypomethylation in open sea regions and hypermethylation within CGIs (Figure 4C).

To understand the regulatory implications of these methylation changes, we performed motif enrichment analysis of differentially methylated positions (DMPs) using HOMER. Hypomethylated regions showed significant enrichment for AP‐1, KLF, and p63 binding motifs (Figure 4D). Notably, AP‐1 and KLF motifs were particularly enriched in hypomethylated promoter‐proximal regions (TSS, 5'UTR, and first exon), suggesting direct influence on transcriptional regulation (Figure S4C). Conversely, hypermethylated regions showed enrichment for SPDEF, NFI, and SOX motifs, accompanied by reduced expression of NFI family members *NFIB* and *NFIX*, which are essential for normal development and have established tumor‐suppressive functions across multiple cancer types (Figure 4D; Figure S4D). These findings aligned with TF enrichment patterns observed in our single‐nucleus chromatin accessibility analysis (Figure 3E; Figure S4E), suggesting that factors such as FOSL2 and JUNB may serve as key mediators of the broader epigenetic reprogramming in SNSCC.

### 2.7 Integration of DNA Methylation and Chromatin Accessibility Reveals Epigenetic Control of Gene Expression

To elucidate the relationship between DNA methylation and transcriptional regulation, we integrated DMPs with DEGs, identifying 129 hypomethylated‐upregulated (hypo‐up) and 102 hypermethylated‐downregulated (hyper‐down) genes containing one or more differentially methylated CpG sites (Figure 4E; Figure S4F). Analysis of the 264 differentially methylated CpG sites associated with hypo‐up genes revealed distinct genomic distributions: 110 hypomethylated sites near 66 genes in promoter‐proximal regions and 154 sites associated with 63 genes in distal regulatory regions (Figure 4F). Motif analysis of hypomethylated promoter‐proximal regions revealed significant enrichment of AP‐1 and p53 family binding motifs (Figure S4G).

Given the established relationship between DNA methylation, chromatin accessibility, and transcriptional activation, we performed an integrative analysis of promoter‐hypomethylated genes and DAR‐linked genes. This analysis identified 19 genes showing concordant epigenetic alterations, with the majority demonstrating elevated expression in tumor epithelial cells (Figure 4G). These genes included key regulators of hypoxic response (*ADM*, *ENO1*, and *NDRG1*) and epithelial development (*DSG3*, *KRT6A*, and *SFN*), all showing significant promoter hypomethylation in SNSCC (Figure 4H). Representative analysis of *ENO1* and *KRT6A* demonstrated that their elevated expression in tumor epithelium corresponded with increased chromatin accessibility and reduced DNA methylation at promoter regions (Figure 4I). These findings establish that coordinated epigenetic reprogramming, particularly in tumor epithelium, drives the transcriptional changes characteristic of SNSCC progression.

### 2.8 Single‐Cell Analysis Reveals Distinct Malignant States and Clinical Relevance

**FIGURE 5:**
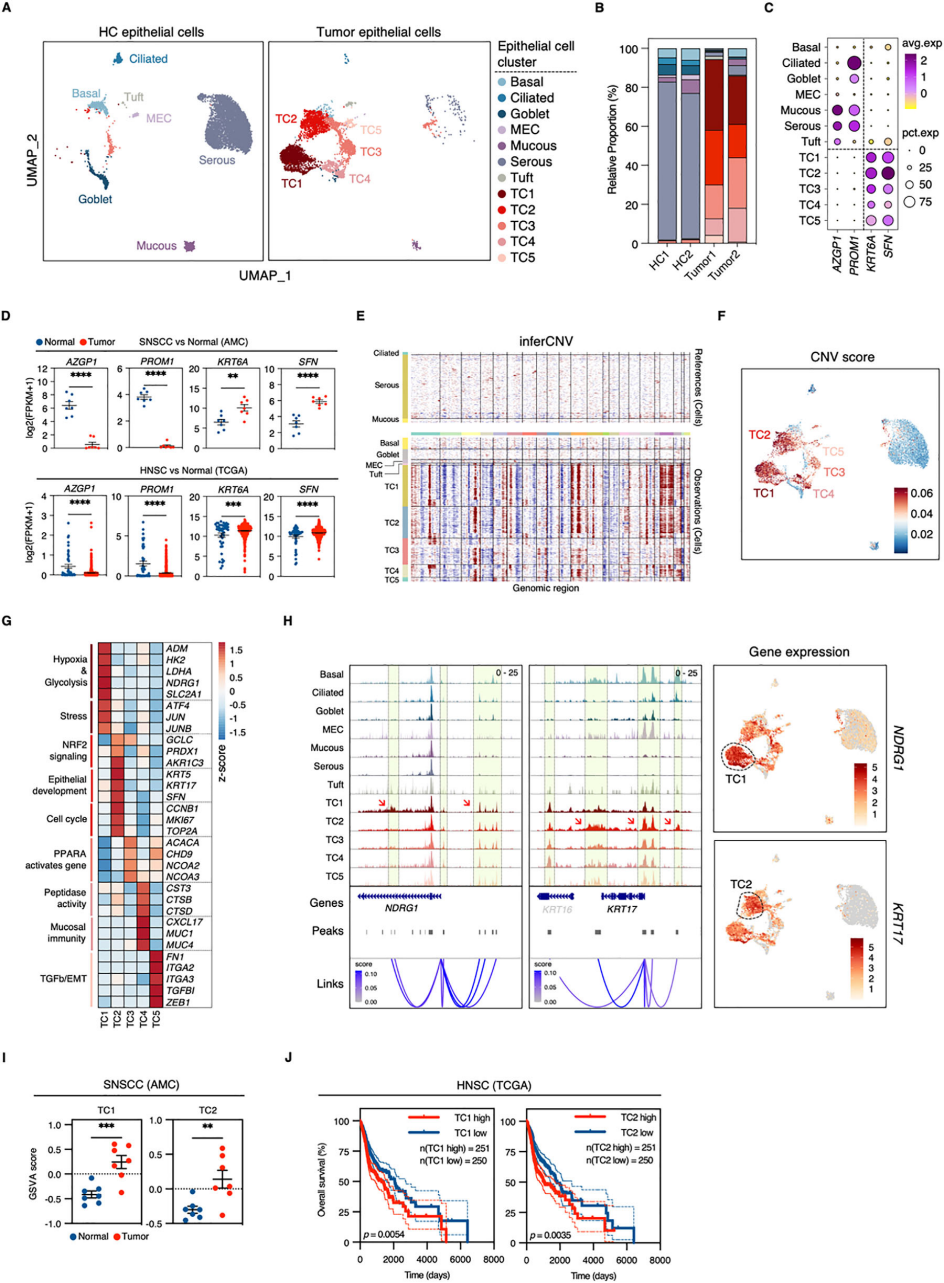
Molecular and phenotypic characterization of distinct malignant epithelial subpopulations in SNSCC. (A) UMAP visualization of epithelial populations in SNSCC and HC samples based on WNN analysis. MEC indicates myoepithelial cells. (B) Quantitative distribution of epithelial cell states across tumor and HC samples. (C) Expression patterns of selected downregulated (*AZGP1* and *PROM1*) and upregulated (*KRT6A* and *SFN*) genes across epithelial subpopulations. (D) Comparative analysis of *AZGP1*, *PROM1*, *KRT6A*, and *SFN* expression in SNSCC versus normal tissues (Upper) and The Cancer Genome Atlas (TCGA) head and neck squamous cell carcinoma (HNSC) dataset (Lower). Data represent mean ± SEM; statistical significance determined by two‐tailed unpaired *t*‐test (*p < 0.05, **p < 0.01, ***p < 0.001, ****p < 0.0001). (E) InferCNV analysis of chromosomal alterations across epithelial subpopulations, using normal mucosa‐enriched ciliated, mucous, and serous cells as reference populations. (F) UMAP projection of epithelial populations colored by inferred CNV scores. (G) Heatmap displaying expression patterns and functional annotations of DEGs across malignant subclusters TC1‐5. (H) Cell type‐specific ATAC‐seq signal profiles for *NDRG1* and *KRT17* regulatory regions (Left) and corresponding UMAP visualization of gene expression (Right). (I) TC1 and TC2 signature scores in SNSCC versus normal samples from bulk RNA‐seq. Data represent mean ± SEM; statistical significance determined by two‐tailed unpaired *t*‐test (*p < 0.05, **p < 0.01, ***p < 0.001, ****p < 0.0001). (J) Kaplan–Meier analysis of overall survival in TCGA HNSC cohort stratified by TC1 and TC2 signature scores (50th percentile cutoff); statistical significance determined by log‐rank test. TC1 signature (HR = 1.45; 95% CI = 1.11–1.90); TC2 signature (HR = 1.48; 95% CI = 1.14–1.93).

Focused analysis of the epithelial compartment identified 15 distinct clusters representing 12 cell types defined by characteristic marker expression (Figure 5A; Figure S5A,B). These populations included basal (*KRT15* and *TP63*), ciliated (*FOXJ1* and *PIFO*), goblet (*MUC5AC* and *SCGB1A1*), myoepithelial (*ACTA2* and *MYH11*), mucous (*MUC5B* and *BPIFB2*), serous (*LYZ* and *LTF*), and tuft cells (*POU2F3* and *TRPM5*), along with five tumor cell subpopulations (TC1‐5) predominantly derived from malignant tissue (Figure 5A,B; Figure S5C,D). In this single‐cell investigation of this rare disease, these five subclusters represent the observed tumor cellular heterogeneity of the analyzed samples. While most epithelial populations were present in HC samples, TC subpopulations and tuft cells showed tumor‐specific enrichment, with only TC3 showing limited representation in control tissue (Figure S5D). TC1‐4 were detected across multiple tumor samples, whereas TC5 demonstrated patient‐specific expression patterns (Figure 5B).

Analysis of gene expression patterns revealed broad expression of established head and neck squamous cell carcinoma (HNSC) markers (*KRT6A* and *SFN*) across tumor subpopulations, while genes typically downregulated in HNSC (*AZGP1* and *PROM1*) showed preferential expression in normal epithelial compartments (Figure 5C,D). Scoring analysis demonstrated significant enrichment of tumor‐associated DEGs across TC populations, particularly in TC1 and TC2, with control tissue‐enriched populations showing inverse patterns (Figure S5E). Copy number variation (CNV) analysis, using normal epithelial populations as references, confirmed the malignant identity of TC clusters, with TC1, TC2, and TC4 demonstrating the highest levels of genomic aberration (Figure 5E,F). These findings establish TC subclusters as distinct malignant populations within the SNSCC epithelial compartment.

Comparative analysis of gene expression profiles across TC clusters revealed distinct functional signatures characterizing each subpopulation (Figure 5G; Figure S5F,G). TC1 demonstrated significant enrichment in hypoxia, glycolysis, and stress response pathways, with elevated expression of key regulators (*ATF4*, *JUN*, and *JUNB*). TC2 showed enrichment in epithelial development (*KRT5*, *KRT17*, and *SFN*), cell cycle regulation (*CCNB1*, *MKI67*, and *TOP2A*), and NRF2 pathway components. TC3 exhibited upregulation of PPARA‐regulated transcripts (*NCOA2* and *NCOA3*) and DNA damage response genes, while TC4 showed enrichment in peptidase activity (*CST3*, *CTSB*, and *CTSD*) and mucosal immunity. TC5 displayed characteristics of epithelial–mesenchymal transition (EMT), including elevated expression of EMT markers (*FN1* and *ZEB1*) and integrins (*ITGA2* and *ITGA3*) (Figure 5G; Figure S5G).

Analysis of chromatin accessibility patterns revealed distinct regulatory landscapes across TC populations compared to normal epithelium (Figure 5H). TC‐specific regulatory elements showed increased accessibility at the *NDRG1* locus across all TC subclusters, with particularly high accessibility in TC1, consistent with the corresponding gene expression pattern. Similar patterns were observed at the *KRT17* locus, with TC2 showing selective enhancement of accessibility at specific regulatory regions, in line with its subcluster‐specific expression (Figure 5H). Integration of TC subcluster‐specific genes with bulk RNA‐seq data demonstrated particular enrichment of TC1 and TC2 signatures among SNSCC‐upregulated genes (Figure S5H). These signatures showed significant elevation in tumor tissues (Figure 5I; Figure S5I), suggesting their widespread presence and potential functional significance. To validate the biological relevance of these signatures beyond our cohort, we compared them with established HNSC transcriptional programs [38]. We observed a striking molecular conservation: TC1 showed strong concordance with hypoxia/stress programs, TC2 with cell cycle, and TC5 with partial‐EMT signatures (Figure S5J). Based on this shared molecular architecture and given that SNSCC is a classified subset within the broader category of HNSC, we assessed the prognostic relevance of these signatures using the most extensive publicly available resource, The Cancer Genome Atlas (TCGA) HNSC cohort. Notably, high expression of TC1 and TC2 signatures correlated with poor survival outcomes in the TCGA HNSC cohort (Figure 5J). Conversely, the signatures for TC3, TC4, and TC5 showed no statistically significant prognostic value in the same cohort (Figure S5K).

### 2.9 Hypoxic Tumor Cells Orchestrate Angiogenic Signaling Networks

**FIGURE 6:**
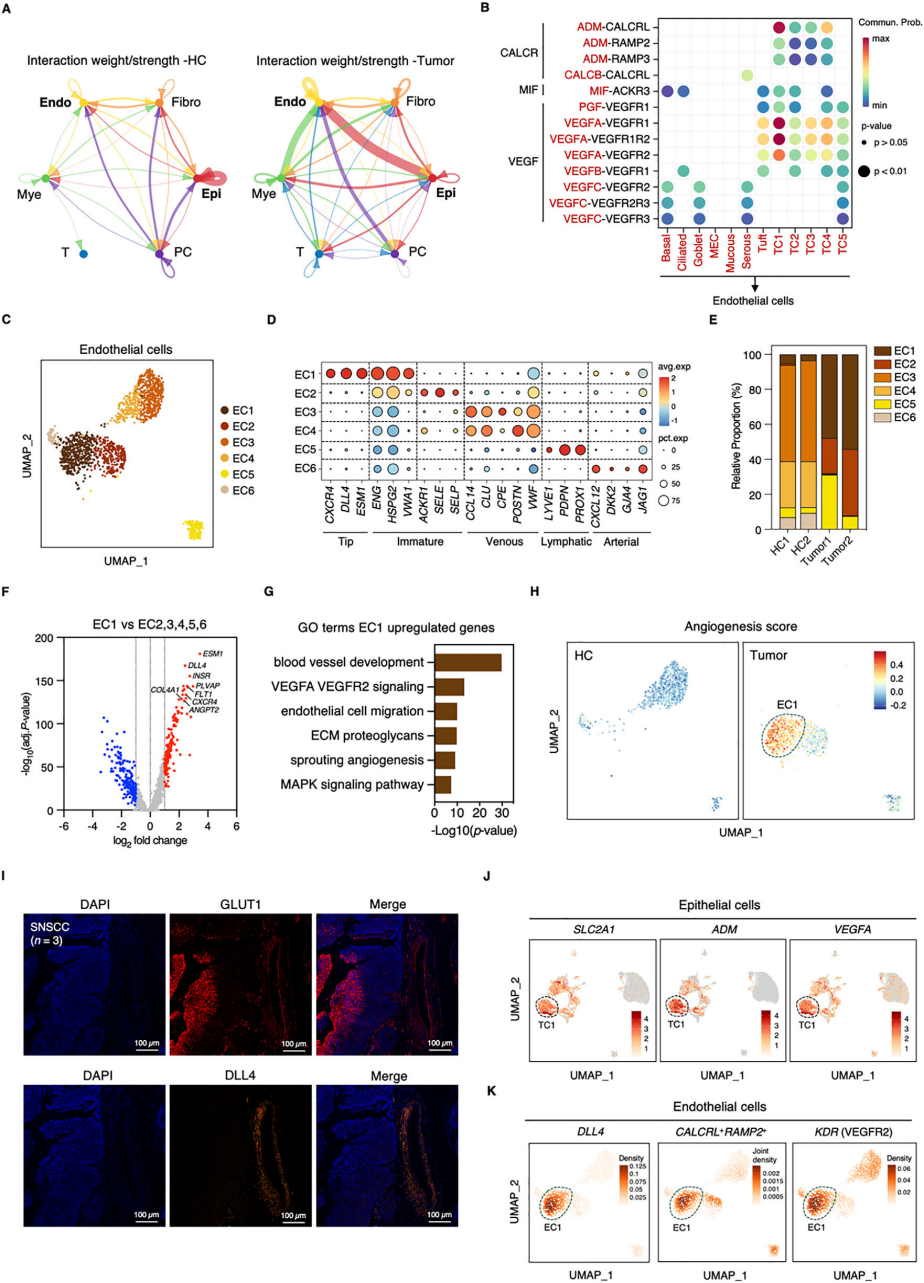
Analysis of tumor–stromal interactions reveals hypoxic tumor cell‐mediated angiogenic signaling in SNSCC. (A) Cell–cell communication networks among major cell populations in SNSCC and HC samples, analyzed using CellChat. Network edges represent secreted signaling interactions; edge thickness is proportional to interaction strength. (B) Quantification of ligand–receptor signaling probabilities for CALCR, MIF, and VEGF pathways from epithelial subpopulations to endothelial cells. (C) UMAP visualization of endothelial populations in SNSCC and HC samples based on WNN analysis. (D) Expression patterns of subpopulation‐specific marker genes across endothelial cell states. (E) Quantitative distribution of endothelial cell states across tumor and HC samples. (F) Differential gene expression analysis comparing EC1 to other endothelial subclusters. Significant DEGs defined by |log2 fold change| > 1 and adjusted *p* < 0.05. Nonsignificant genes are shown in gray. (G) GO terms enriched in upregulated genes from the EC1 population identified in (F). (H) UMAP projection of angiogenesis signature scores across endothelial populations. (I) Histological evidence of tumor–endothelial cell interaction in patient SNSCC tissues (*n =* 3). Immunofluorescence (IF) staining shows co‐localization of GLUT1 (red, expressed on hypoxic tumor cells; upper panels) and DLL4 (orange, expressed on endothelial tip cells; lower panels), merged with DAPI (blue, nuclei). Representative images are shown. Main panel scale bar = 100 µm. (J) UMAP visualization of *SLC2A1*, *ADM*, and *VEGFA* expression across epithelial compartments. (K) Density plots of *DLL4* ^+^, *CALCRL* ^+^ *RAMP2* ^+^, and *KDR* ^+^ cells within endothelial compartments.

Analysis of cell–cell communication networks revealed enhanced interactions between epithelial and endothelial populations in tumor tissue compared to HCs (Figure 6A). This observation aligned with significant upregulation of hypoxia and VEGF signaling pathways identified in bulk transcriptome analysis (Figure S6A). Given the established role of hypoxic microenvironments in promoting aggressive tumor phenotypes, and building upon our finding that the clinically relevant TC1 epithelial population exhibits strong hypoxia and glycolysis signatures (Figure 5G; Figure S6B) and correlates with poor patient survival (Figure 5J), we focused our subsequent analysis on secreted factors originating from this specific hypoxic population.

Investigation of epithelial‐to‐endothelial signaling revealed enrichment of CALCR, MIF, and VEGF pathways in tumor tissue, with particularly strong signals originating from TC1 populations (Figure S6C,D). Conversely, WNT signaling showed enrichment in control tissue, primarily originating from plasma cells (Figure S6C,D). Beyond interactions with endothelial cells, MIF signaling was also found to be strongly activated between both TC1 and TC2 clusters and myeloid cells, suggesting potential involvement in pro‐tumor myeloid activation within TME (Figure S6D). Analysis of specific ligand–receptor interactions identified ADM‐CALCRL, ADM‐RAMP2, ADM‐RAMP3, MIF‐ACKR3, VEGFA‐VEGFR1, and VEGFA‐VEGFR2 as predominant signaling axes between TC1 and endothelial cells, providing molecular candidates driving the enhanced epithelial–endothelial crosstalk (Figure 6B).

### 2.10 Tumor‐Specific Endothelial Populations Drive Angiogenic Programming

Given the pronounced enhancement of epithelial‐to‐endothelial interactions in tumor tissue (Figure 6A) and the identification of specific mediating ligand–receptor pairs targeting the endothelial compartment (Figure 6B), we next performed a detailed analysis of the endothelial cell compartment to investigate its cellular composition and heterogeneity in the TME.

Characterization of endothelial heterogeneity revealed six distinct clusters within SNSCC and HC tissues, following exclusion of doublet populations (Figure 6C; Figure S6E,F). Expression analysis of canonical markers defined five endothelial subtypes: tip cells (*CXCR4*, *DLL4*, and *ESM1*), immature cells (*ENG*, *HSPG2*, and *VWA1*), venous cells (*CLU* and *VWF*), lymphatic cells (*LYVE1* and *PROX1*), and arterial cells (*CXCL12* and *GJA4*) (Figure 6D). Immature populations demonstrated concurrent expression of stalk‐like markers (*ACKR1*, *SELE*, and *SELP*), while venous cells comprised two transcriptionally similar subsets. Notably, EC1 (tip) and EC2 (immature) clusters showed tumor‐specific enrichment, contrasting with the preferential venous cell enrichment in healthy tissue (Figure 6E).

Differential expression analysis comparing EC1 and EC2 to other endothelial populations revealed distinct transcriptional programs (Figure 6F; Figure S6G). GO analysis demonstrated enrichment of angiogenic processes in tumor‐associated populations (Figure 6G; Figure S6H). EC1 showed particular enrichment in angiogenesis‐related pathways and extracellular matrix regulation, with elevated expression of angiogenic regulators (*ANGPT2* and *PLVAP*), matrix components (*COL4A1* and *COL4A2*), and integrins (*ITGA1* and *ITGA8*). In contrast, EC2 demonstrated enrichment in oxygen response (*HIF1A* and *NDRG1*) and immune‐related programs (*ACKR1*, *SELP*, *NFKBIA*, and *ICAM1*) (Figure 6G; Figure S6H,I). Consistent with these profiles, EC1 exhibited the highest angiogenic signature scores (Figure 6H).

Intercellular communication analysis quantified interactions between epithelial and endothelial subtypes, revealing particularly robust connections between the TC1 epithelial and EC1 endothelial clusters. Detailed analysis of ADM signaling originating from TC1 cells, mediated by the CALCRL/RAMP receptor complex, revealed both broad engagement via ADM‐CALCRL with multiple endothelial populations and a pronounced, selective enrichment of ADM‐RAMP2 interactions with the EC1 cluster (Figure S6J). Similarly, the VEGFA‐VEGFR2 axis also constituted a key signaling pathway mediating TC1‐EC1 crosstalk, exhibiting robust engagement with the EC1 population (Figure S6J). These findings establish that hypoxic tumor cells coordinate angiogenic responses through multiple secreted factors acting on tumor‐associated endothelial cells.

To further investigate the spatial and cellular underpinnings of these angiogenic signaling components, we performed immunofluorescence (IF) and leveraged our single‐cell expression analyses. To enhance functional specificity, the key hypoxic cell population (TC1) was marked by GLUT1 (*SLC2A1*), and the angiogenic endothelial tip cell population (EC1) was marked by DLL4. IF analysis demonstrated robust spatial proximity and co‐enrichment of GLUT1‐positive tumor regions and DLL4‐positive endothelial structures (Figure 6I). We performed quantitative analysis to measure the spatial co‐enrichment of these two functional markers across the three patient samples (Figure S6K). This spatial arrangement supports a complex network of hypoxia‐induced ligand–receptor interactions directly driving tumor angiogenesis. Complementing these spatial observations, single‐cell expression data revealed that pro‐angiogenic factors and hypoxia‐induced genes (*SLC2A1, ADM*, and *VEGFA*) showed significant elevation in the TC1 population (Figure 6J). Density plots further indicated that *DLL4*, ADM receptors (*CALCRL* ^+^ *RAMP2* ^+^), and *KDR* (VEGFR2) were predominantly high in the EC1 endothelial cell cluster (Figure 6K). These integrated results delineate the cell type‐specific localization of pro‐angiogenic ligands and their corresponding receptors, thereby establishing the fundamental architectural elements driving the TC1‐EC1 axis in SNSCC.

### 2.11 Hypoxia Induces Pro‐Angiogenic Factors in Tumor Cells and Shapes the TME

**FIGURE 7:**
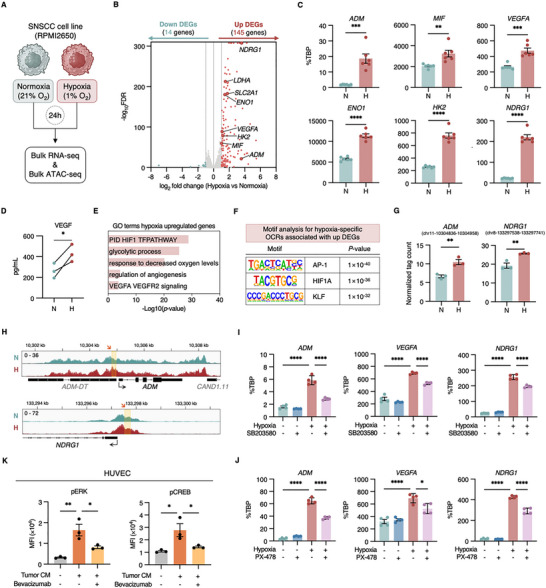
Hypoxia‐induced transcriptomic and epigenomic reprogramming drives pro‐angiogenic signaling in SNSCC cells. (A) The human nasal squamous cell carcinoma cell line, RPMI 2650, was subjected to either normoxia (21% O_2_) or hypoxia (1% O_2_) for 24 h. Subsequently, cells were harvested for bulk RNA‐seq and bulk ATAC‐seq analyses to characterize transcriptomic changes and chromatin accessibility, respectively (Created in BioRender. BioRender.com/lt2jloc). (B) Volcano plot of bulk RNA‐seq data from RPMI 2650 cells, displaying transcriptomic changes between normoxic and hypoxic conditions. Colored dots indicate genes with |log2 fold change| > 1, adjusted *p* < 0.05, and FPKM > 1. Nonsignificant genes are shown in gray. (C) Validation of gene expression for *ADM*, *MIF*, *VEGFA*, *ENO1*, *HK2*, and *NDRG1* in RPMI 2650 cells under varying oxygen conditions by RT‐qPCR. Expression levels were normalized to *TBP*. Data represent mean ± SEM; statistical significance determined by two‐tailed unpaired *t*‐test (*p < 0.05, **p < 0.01, ***p < 0.001, ****p < 0.0001) (D) Concentrations of VEGF in conditioned medium (CM) from RPMI 2650 cells, cultured under normoxic and hypoxic conditions, measured by enzyme‐linked immunosorbent assay (ELISA). Data represent mean ± SEM from three independent experiments; statistical significance determined by two‐tailed paired *t*‐test (*p < 0.05, **p < 0.01, ***p < 0.001, ****p < 0.0001) (E) GO enrichment analysis of DEGs significantly upregulated in RPMI 2650 cells under hypoxic conditions compared to normoxia. (F) TF binding motifs significantly enriched in hypoxia‐specific open chromatin regions (OCRs) associated with upregulated DEGs were identified through *de novo* motif analysis. (G) Quantification of ATAC‐seq tag counts at specific OCRs within the *ADM* and *NDRG1* loci in RPMI 2650 cells under hypoxic and normoxic conditions. Values represent normalized tag counts (mean ± SEM from *n* = 3 technical replicates); statistical significance determined by two‐tailed unpaired *t*‐test (**p < 0.01). (H) ATAC‐seq tracks displaying enhanced signal at the *ADM* and *NDRG1* loci in RPMI 2650 cells under hypoxic (red) compared to normoxic (green) conditions. The highlighted regions (orange boxes) correspond to the specific OCRs shown in (G). (I) Validation of gene expression for *ADM*, *VEGFA*, and *NDRG1* in RPMI 2650 cells under normoxic or hypoxic conditions, with or without treatment with SB203580. Data represent mean ± SEM; statistical significance determined by one‐way ANOVA (*p < 0.05, **p < 0.01, ***p < 0.001, ****p < 0.0001). (J) Validation of gene expression for *ADM*, *VEGFA*, and *NDRG1* in RPMI 2650 cells under normoxic or hypoxic conditions, with or without treatment with PX‐478. Data represent mean ± SEM; statistical significance determined by one‐way ANOVA (*p < 0.05, **p < 0.01, ***p < 0.001, ****p < 0.0001). (K) Functional in vitro signaling analysis in HUVECs. Phosphorylation levels of ERK and CREB in HUVECs treated with hypoxic tumor CM with or without the VEGF neutralizing antibody, Bevacizumab. Geometric mean fluorescence intensity (gMFI) values are shown. Data represent mean ± SEM; statistical significance determined by one‐way ANOVA (*p < 0.05, **p < 0.01, ***p < 0.001, ****p < 0.0001). Three independent experiments were performed (*n* = 3).

In light of the established role of the hypoxic TME in promoting angiogenesis and the involvement of specific epithelial populations (e.g., TC1) in this process, we sought to empirically test and mechanistically dissect how hypoxia directly impacts tumor cells to induce the expression of pro‐angiogenic factors. To elucidate these mechanisms, we exposed the SNSCC cell line (RPMI 2650) to normoxic (21% O_2_) or hypoxic (1% O_2_) conditions for 24 h and performed parallel RNA‐seq and ATAC sequencing (ATAC‐seq) analyses (Figure 7A).

PCA of the bulk RNA‐seq data revealed a clear separation between normoxic and hypoxic tumor samples, confirming the significant impact of hypoxia on the global transcriptome (Figure S7A). Transcriptomic profiling revealed substantial gene expression alterations, with 145 upregulated and 14 genes downregulated under hypoxic conditions (adjusted *p*‐value < 0.05, |log2 fold change| > 1) (Figure 7B). Notably, genes significantly upregulated by hypoxia included key factors previously implicated in epithelial–endothelial interactions and angiogenesis, such as *ADM, MIF*, and *VEGFA*, alongside metabolic and hypoxia‐response genes including *ENO1, HK2, NDRG1, LDHA*, and *SLC2A1* (Figure 7B). RT‐qPCR validation confirmed the upregulation of these genes in tumor cells under hypoxic conditions, corroborating bulk RNA‐seq results (Figure 7C). Further empirical support came from enzyme‐linked immunosorbent assay (ELISA), which confirmed increased VEGF protein levels in hypoxic cell line supernatants, demonstrating the hypoxia‐induced production of this key angiogenic factor (Figure 7D). GO analysis of hypoxia‐upregulated genes demonstrated significant enrichment for biological programs related to VEGFA‐VEGFR2 signaling, regulation of angiogenesis, response to decreased oxygen levels, and glycolytic processes (Figure 7E). Collectively, these in vitro findings indicate that hypoxia directly induces the expression of pro‐angiogenic and metabolic genes in SNSCC tumor cells, providing a mechanistic basis for the observed angiogenic programs.

To investigate the transcriptional regulatory mechanisms by which hypoxia upregulates these genes, we performed ATAC‐seq analysis. PCA of the ATAC‐seq data demonstrated a distinct segregation of normoxic and hypoxic samples (Figure S7B). Motif enrichment analysis of hypoxia‐specific open chromatin regions (OCRs) associated with upregulated genes identified significant enrichment of binding motifs for AP‐1, HIF1A, and KLF family members (Figure 7F), indicating that these TFs likely mediated the hypoxic response. Genome browser tracks of representative OCRs demonstrated increased chromatin accessibility at hypoxia‐responsive gene loci, including *ADM, NDRG1*, *ENO1*, and *HK2*, under hypoxic conditions (Figure 7G,H; Figure S7C,D).

To functionally validate the upstream role of the identified TFs in driving the hypoxic program, we treated SNSCC cells with selective inhibitors. Inhibition of AP‐1 signaling using the p38 MAPK inhibitor SB203580 and HIF1A inhibition with PX‐478 significantly attenuated the hypoxia‐induced expression of pro‐angiogenic factors (e.g., *ADM*, *VEGFA*, and *MIF*) and core hypoxia‐response genes (e.g., *NDRG1* and *HK2*) (Figure 7I,J; Figure S7E,F). This confirms that both AP‐1 and HIF1A activities are essential for regulating the major angiogenic and hypoxia‐driven transcriptional programs in SNSCC.

Linking the hypoxic transcriptional output to the biological function, we performed in vitro functional studies using Human Umbilical Vein Endothelial Cells (HUVECs). To verify the ability of hypoxic TC1 cells to induce angiogenesis, we treated HUVECs with hypoxic tumor conditioned medium (CM). The phosphorylation of ERK (pERK) and CREB (pCREB) was significantly induced by CM in HUVECs (Figure 7K). Since TC1 cells co‐express both *ADM* and *VEGFA*, we tested the contribution of VEGFA to this potent signaling cascade. Tumor CM treatment‐induced activation was significantly attenuated by the VEGF neutralizing antibody, Bevacizumab, confirming that VEGFA is a critical factor in the TC1‐induced angiogenic signaling (Figure 7K). Moreover, ADM treatment was sufficient to significantly increase pERK levels in HUVECs, demonstrating ADM's capacity to trigger the angiogenic signaling pathway (Figure S7G).

To confirm the clinical relevance of genes upregulated by hypoxia, we assessed their expression in human tumors. Comparison of bulk RNA‐seq data from TCGA HNSC tumors with that from normal tissues and RT‐qPCR analysis of paired SNSCC and normal tissues revealed significantly elevated expression of key hypoxia‐induced pro‐angiogenic genes (e.g., *ADM, MIF*, and *VEGFA*) in the tumors, underscoring the clinical relevance of our in vitro findings (Figure S7H,I).

We further investigated ADM, a hypoxia‐induced angiogenic factor, and found significant hypomethylation at the *ADM* locus (Figure S7J) and increased chromatin accessibility in tumor epithelial cells (Figure S7K), indicating epigenetic regulation of *ADM* expression in SNSCC. Analysis of the TCGA HNSC cohort demonstrated that *ADM* expression progressively increased with advancing clinical stage and was significantly elevated in tumors with lymphovascular invasion and in patients with poor overall survival (Figure S7L). *ADM* expression also showed significant correlation with *VEGFA* expression (Figure S7M) and was associated with poor overall survival outcomes in HNSC patients (Figure S7N).

These findings demonstrate that hypoxic tumor cells, particularly the TC1 population, coordinate angiogenic responses through secretion of multiple pro‐angiogenic factors, with ADM and VEGFA representing a potential therapeutic target in SNSCC progression.

## 3 Discussion

The molecular underpinnings of SNSCC have remained poorly understood due to its rarity and the limited availability of patient samples for comprehensive analysis. Our integrative multi‐omic profiling reveals comprehensive molecular characteristics and cell type‐specific signatures of the SNSCC landscape. Through the integration of bulk and single‐cell approaches, we identified core oncogenic programs, epigenetic regulation of transcriptional changes, and heterogeneous cellular components driving SNSCC pathogenesis.

A central finding of our study is the identification of distinct malignant cell populations with clinical relevance. The hypoxic (TC1) and proliferative (TC2) clusters not only define the cellular heterogeneity within SNSCC but also highlight how different tumor cell states may contribute to disease progression. The strong association between these populations and poor clinical outcomes in the HNSC dataset suggests they may represent key drivers of aggressive disease behavior. It must be noted that due to the rarity of SNSCC, the initial single‐nucleus analysis was based on a limited number of samples, rendering the derived TC1–TC5 subclusters and signatures exploratory and hypothesis‐generating. Furthermore, while SNSCC is a subset of HNSC, the prognostic correlation should be interpreted cautiously, as reflecting a shared aggressive SCC phenotype rather than a definitive, generalizable SNSCC‐specific prognostic indicator. Particularly noteworthy is the TC1 cluster's hypoxic signature. These hypoxic features are prominently represented among tumor‐upregulated genes compared to normal mucosa in our bulk transcriptomic data. The robust glycolytic programming in TC1 cells likely represents an adaptation to sustained hypoxic stress, enabling these cells to survive in nutrient‐poor conditions while maintaining aggressive phenotypes. This finding has important therapeutic implications, as hypoxic tumor cells are often resistant to conventional treatments. The co‐existence of TC1 and TC2 populations suggests that therapeutic strategies targeting either population alone may be insufficient, supporting the development of combination approaches that address both hypoxic and proliferative tumor compartments.

Our discovery of the TC1‐EC1 signaling axis reveals a previously unrecognized mechanism driving tumor angiogenesis in SNSCC. The preferential interaction between hypoxic tumor cells (TC1) and endothelial tip cells (EC1), mediated through hypoxia‐induced factors including ADM, MIF, and VEGFA, represents a key mechanism for tumor‐driven angiogenesis in SNSCC. Our IF analyses provided direct histological and critical spatial evidence supporting this cellular crosstalk. Specifically, we demonstrated significant co‐enrichment of functionally specific markers GLUT1 (a hypoxic tumor marker) with DLL4 (an endothelial tip cell marker) at the tumor‐endothelial interface. This spatial proximity suggests a potential localized communication, where factors secreted by hypoxic tumor cells may influence adjacent endothelial cells to initiate pro‐angiogenic events. However, we acknowledge that quantitative IF analysis across the limited patient samples (*n* = 3) revealed patient‐to‐patient variability (Figure S6K); thus, this finding is interpreted as strong mechanistic evidence supporting the cellular crosstalk, recognizing that establishing statistical generalizability requires larger SNSCC tissue cohorts. The identification of ADM and VEGFA as co‐regulatory mediators in this axis is particularly significant, suggesting opportunities for dual therapeutic disruption of tumor vasculature. ADM expression is associated with poor clinical outcomes in HNSC patients, suggesting its potential relevance in aggressive disease progression.

The pivotal role of ADM in the SNSCC angiogenic axis aligns with its established significance in cancer progression. ADM is a recognized pro‐angiogenic peptide, notably induced by hypoxia, contributing to carcinogenesis and progression [39]. In vitro studies show that ADM promotes capillary‐like tube formation and activates signaling pathways, including Akt, in endothelial cells [40]. Furthermore, ADM secreted from hypoxic tumor cells induces capillary morphogenesis in HUVECs [41] and directly promotes endothelial proliferation, migration, and tube formation [42]. These observations are consistent with recent findings in other malignancies, such as melanoma, where ADM drives not only angiogenesis but also lymphangiogenesis and metastasis [43]. Crucially, our single‐nucleus analysis reveals that endothelial tip cells (EC1) in SNSCC predominantly express RAMP2. Given that RAMPs dictate the specific signaling bias of ADM toward proliferative pathways [44], this specific receptor configuration likely amplifies the angiogenic potency of TC1‐derived ADM. Additionally, recent evidence suggests ADM may also remodel the immune microenvironment by suppressing endothelial CCL2 secretion [45], highlighting the potential for targeting this axis to simultaneously disrupt angiogenesis and restore antitumor immunity.

To sustain this potent angiogenic phenotype, our study reveals that TC1 cells utilize a robust upstream transcriptional and epigenetic program to coordinately regulate *ADM* and *VEGFA* expression. Specifically, by integrating bulk RNA‐seq and ATAC‐seq profiles of hypoxic SNSCC cells, we identified a distinct epigenetic reprogramming event, characterized by increased chromatin accessibility at regulatory elements enriched for AP‐1 and HIF1A binding motifs. While *VEGFA* is a classical hypoxia target, our functional studies using selective inhibitors (SB203580 for p38 MAPK‐mediated AP‐1 signaling and PX‐478 for HIF1A) confirm that the high‐level co‐expression of *ADM* and *VEGFA* is dependent on the cooperative action of these two TFs. This establishes AP‐1 not merely as a bystander, but as a critical co‐regulator that reinforces the HIF1A‐driven hypoxic response, thereby locking TC1 cells into a highly angiogenic state. Furthermore, the biological consequence of this TC1 transcriptional output was functionally confirmed in vitro: hypoxic tumor CM significantly induced pERK and pCREB activation in HUVECs. This angiogenic signaling is regulated by both VEGFA (attenuated by Bevacizumab) and ADM (independent single‐treatment efficacy), underscoring the necessity of targeting this dual‐factor axis in SNSCC.

The epigenomic landscape of SNSCC demonstrates complex regulatory mechanisms that extend beyond previously reported DNA methylation patterns [19]. Through integrated analysis of chromatin accessibility and DNA methylation states, we identified cell type‐specific regulatory programs that distinguish tumor‐associated populations from their normal counterparts. The DNA methylation analysis provides essential epigenetic validation for our single‐cell findings. The integrated approach confirms that the dynamic transcriptional changes revealed by snRNA/ATAC‐seq are rooted in stable epigenetic control. Specifically, key genes showing transcriptional changes in the malignant clusters exhibited direct functional concordance with significant hypo‐methylation at their promoter regions. The consistent enrichment of AP‐1 motifs in both hypomethylated regions and accessible chromatin across multiple cell types suggests AP‐1 transcription factors serve as master regulators of the SNSCC cellular phenotype. This finding has immediate therapeutic relevance given recent advances in developing small‐molecule inhibitors targeting AP‐1 family members [46, 47].

Given these pro‐tumorigenic effects, targeting ADM signaling presents a compelling therapeutic strategy. Multiple studies demonstrate that ADM inhibition reduces tumor growth and impairs angiogenic processes by limiting endothelial cell activation or normalizing tumor vasculature [40, 41, 48]. Notably, recent findings indicate that ADM secreted by hypoxic TAMs destabilizes endothelial cell adherens junctions, suggesting that ADM blockade may restore vascular integrity and improve drug delivery [48]. The identification of ADM as a critical TC1‐derived mediator in the SNSCC angiogenic axis provides a strong rationale for its direct therapeutic targeting. While our functional studies highlighted the necessity of also addressing VEGFA (via Bevacizumab), ADM remains a novel and specific candidate for intervention due to its unique epigenetic regulation and its independent capacity to activate HUVEC signaling. These findings collectively highlight the translational potential of ADM inhibition as a strategy to impair tumor progression by targeting pathological angiogenesis. In this context, targeting the hypoxia–angiogenesis axis, mediated primarily by ADM and VEGFA, represents a promising therapeutic approach, particularly for SNSCC patients characterized by a pronounced hypoxic signature.

The immune landscape of SNSCC demonstrates distinctive features, particularly substantial infiltration of myeloid cells expressing tumor‐promoting pathways. These findings align with previous observations of increased myeloid cell proportions in recurrent cases [49], suggesting that microenvironmental composition may critically influence disease progression. In this context, recent work by Benmebarek et al. is highly pertinent, demonstrating that anti‐VEGF treatment, an established strategy for angiogenic modulation, can potentiate immune checkpoint blockade by actively reprogramming the TME to foster antitumor immunity through BAFF‐ and IL‐12‐dependent mechanisms [50]. While single‐nucleus analysis provides limited depth of immune cell characterization, the identified patterns suggest that successful immunotherapeutic strategies may require specific targeting of the myeloid compartment, potentially in conjunction with angiogenic normalization.

These findings have several important therapeutic implications: (i) the identification of distinct tumor subpopulations suggests combination therapies targeting multiple cellular states may be necessary, (ii) characterization of the TC1‐EC1 signaling axis provides a rationale for targeted anti‐hypoxia and anti‐angiogenic approaches through ADM and VEGFA pathway inhibition, and (iii) the identified epigenetic landscapes suggest opportunities for therapeutic modulation of AP‐1‐dependent transcription.

In conclusion, our integrated multi‐omic analysis reveals the complex molecular architecture driving SNSCC progression. We provide a high‐resolution map of the tumor ecosystem, identifying clinically relevant subpopulations (TC1/TC2) and delineating a critical hypoxia‐driven angiogenic axis mediated by ADM and VEGFA. Mechanistically, we show that AP‐1 and HIF1A act as essential regulators, orchestrating the epigenetic and transcriptional reprogramming that fuels this aggressive phenotype. While the prognostic correlations warrant validation in larger SNSCC‐specific cohorts, our findings identify ADM as a promising and immediately targetable node to disrupt epigenetically controlled angiogenesis. Future studies are warranted to further validate the therapeutic efficacy of targeting this axis and to translate these mechanistic insights into effective combination therapies for SNSCC patients.

## 4 Materials and Methods

### 4.1 Patient Sample Collection

Tumor samples were obtained from Korean adult patients with sinonasal squamous cell carcinoma (SNSCC) at the time of surgical resection. Briefly, tumors were resected, flash‐frozen, and stored at a temperature of less than −80°C until RNA and DNA extraction. Each frozen tumor specimen had an accompanying normal tissue from the contralateral nasal cavity. The weight of frozen tumor and normal tissue was at least 150 mg each, and usually less than 200 mg. This study was conducted in accordance with the Declaration of Helsinki and approved by the Institutional Review Board of Asan Medical Center (2019‐0708). All participants provided written informed consent for inclusion before they participated in the study. Similar to clinical populations, all patients were treated with curative intent. All cases were collected regardless of surgical stage or histologic grade and staged according to the eighth edition of the American Joint Committee on Cancer.

Complete clinical data elements were collected, including gender, age at diagnosis, tumor subsite, year of tumor collection, tumor differentiation, smoking and alcohol history, initial treatment modalities, postoperative radiotherapy and/or chemotherapy, and follow‐up data, including recurrence and survival. Time points were calculated from the start date of treatment, and recurrence (a new tumor event) and survival (time to death from SNSCC or other causes or the last follow‐up) were also assessed.

### 4.2 Cell Culture

The human nasal squamous cell carcinoma cell line RPMI 2650 was obtained from the Korean Cell Line Bank. Cells were cultured in RPMI 1640 medium (Gibco, Catalog no. 11875‐093) supplemented with 10% fetal bovine serum (FBS) (Gibco, Catalog no. 16000‐044) and 1% penicillin/streptomycin (Gibco, Catalog no. 15070‐063). Human umbilical vein endothelial cells (HUVECs) were obtained from Gibco (Catalog no. C0035C, Lot no. 2991556). The HUVECs were routinely cultured in Medium 200 (Gibco, Catalog no. M200500) supplemented with Large Vessel Endothelial Supplement (Gibco, Catalog no. A1460801) and utilized for all functional experiments between passages 4 and 7 to ensure consistent functional characteristics. Both cells were maintained in a humidified incubator at 37 °C with 5% CO_2_.

### 4.3 Hypoxia Treatment

To investigate the effects of hypoxia, RPMI 2650 cells were seeded in 12‐well plates at a density of 2 × 10^5^ cells/well. Following 24 h of initial culture under normoxic conditions (37 °C, 5% CO_2_ in a humidified atmosphere), cells were subsequently exposed to either normoxia or hypoxia for an additional 24 h. Hypoxic conditions were established using a hypoxia chamber (Astec), which maintained a humidified atmosphere of 1% O_2_ and 5% CO_2_ at 37 °C.

For inhibitor studies, cells were treated with 10 µm of the p38 MAPK inhibitor SB203580 (MedChemExpress, Catalog no. HY‐10256) or 20 µm of the HIF1A inhibitor PX‐478 (MedChemExpress, Catalog no. HY‐10231) based on distinct time schedules. SB203580 treatment involved a 30 min pre‐treatment followed by 48 h of co‐treatment under the respective normoxic or hypoxic conditions. PX‐478 treatment involved a 24 h pre‐treatment, with co‐treatment continuing for an additional 24 h under the respective conditions.

### 4.4 RNA Extraction

Total RNA was extracted from tissue specimens in accordance with the RNeasy Mini kit (Qiagen, Catalog no. 74104) following the manufacturer's instructions. RNA concentration was quantified by measuring UV absorbance at 260 nm with a spectrophotometer, and RNA integrity was checked using an Agilent Technologies 2100 Bioanalyzer with an RNA integrity number value greater than or equal to 7.0. For RPMI 2650 cells, total RNA was extracted using the Ribospin II kit (GeneAll Biotechnology, Catalog no. 314‐150) following the manufacturer's protocol.

### 4.5 Bulk RNA‐seq Analysis

Following RNA extraction, sequencing libraries were constructed using the Illumina TruSeq RNA Library Prep Kit (Illumina, Catalog no. 20020189) following the manufacturer's guidelines. The raw data were processed by trimming with Trim Galore, and the reads were subsequently aligned to the human reference genome *hg38* using the STAR aligner [51] with default parameters. Gene expression levels in each sample were normalized using fragments per kilobase of transcript per million mapped reads (FPKM). Differential expression analysis was performed using DESeq2 v1.34.0 [52] on raw read counts. For the SNSCC tumors versus normal tissues, genes were considered differentially expressed with an FPKM > 2, |log2 fold change| > 1, and adjusted *p*‐value < 0.05. For hypoxic versus normoxic RPMI 2650 cells, the criteria for differential expression were an FPKM > 1, |log2 fold change| > 1, and adjusted *p*‐value < 0.05.

Functional enrichment of DEGs was performed using Metascape [53] (https://metascape.org), and GSEA was conducted using the hallmark gene set from the Molecular Signatures Database (MsigDB) [54, 55] (https://www.gsea‐msigdb.org/gsea/index.jsp). The PROGENy package v1.16.0 [56] was employed to evaluate the activity of 14 key signaling pathways in each sample.

### 4.6 cDNA Synthesis

For reverse transcription, 0.15 µg of total RNA from tissue specimens and 0.5 µg of total RNA from RPMI 2650 cells were independently reverse transcribed into cDNA using the RevertAid First Strand cDNA Synthesis kit (Thermo Fisher Scientific, Catalog no. K1622) following the manufacturer's instructions.

### 4.7 Real‐Time Quantitative PCR

RT‐qPCR was performed using the TOPreal qPCR 2X PreMIX (SYBR Green with low ROX; Enzynomics Co. Ltd., Catalog no. RT500M). The RT‐qPCR conditions included an initial denaturation cycle at 95 °C for 10 min, followed by 50 cycles of denaturation at 95 °C for 10 s, 60 °C for 15 s (annealing), and 72 °C for 20 s (extension). A melting curve analysis was executed from 72 °C to 95 °C with a heating rate of 1 °C/45 s. Spectral data were captured and analyzed using the Rotor‐Gene Q instrument (v2.3.1; Qiagen). TATA‐box binding protein (*TBP*) was used as a reference gene, and target gene expression was normalized to *TBP* expression. Primer sequences used to assess mRNA expression are provided in Table 3.

**TABLE 3:** Primers used for RT‐qPCR.

Gene	Forward primer (5’ to 3’)	Reverse primer (5’ to 3’)
*TBP*	CCCGAAACGCCGAATATAATCC	AATCAGTGCCGTGGTTCGTG
*ADM*	CATGAACAACTTCCAGGGCC	ACTGGTAGATCTGGTGTGCC
*MIF*	CGCAGAACCGCTCCTACA	GCCGCGTTCATGTCGTAATA
*VEGFA*	AGGGCAGAATCATCACGAAGT	AGGGTCTCGATTGGATGGCA
*ENO1*	GCCGTGAACGAGAAGTCCTG	ACGCCTGAAGAGACTCGGT
*HK2*	TGCCACCAGACTAAACTAGACG	CCCGTGCCCACAATGAGAC
*NDRG1*	CCAACAAAGACCACTCTCCTC	CCATGCCCTGCACGAAGTA

### 4.8 Deconvolution of Bulk RNA‐seq Data

The CIBERSORTx [57] (https://cibersortx.stanford.edu) deconvolution algorithm was utilized to estimate the relative abundances of 22 immune cell types (LM22) using gene expression levels from bulk RNA‐seq performed on tumor and normal tissues of patients with SNSCC.

### 4.9 DNA Methylation Profiling

DNA was extracted from tissue specimens in accordance with the QIAamp DNA Mini (Qiagen, Catalog no. 51304) following the manufacturer's instructions. DNA methylation profiles were generated using the Illumina HumanMethylation EPIC array platform. These profiles were subsequently integrated with publicly accessible DNA methylation EPIC array data of SNSCC tumor and normal samples from the GSE189778 dataset [19]. All DNA methylation data were processed and analyzed using the ChAMP package v2.24.0 [58] in the R environment. Following a pipeline that included filtering of unreliable detections and probes, quality control, normalization, and batch effect removal via the *champ.runCombat* function, we proceeded to identify DMPs. The comparison between SNSCC tumor and normal samples was conducted using the *champ.DMP* function. Significant DMPs were defined by stringent criteria, which included an adjusted *p*‐value < 0.05 and a beta‐value difference exceeding 20% (|Δβ| > 0.2). Motif enrichment analysis of differentially methylated CpG sites was conducted with the HOMER software v4.11.1 [59] utilizing the findMotifsGenome.pl function, with the parameters set to “size 200 ‐mask.”

### 4.10 Single‐Nucleus Multiome Library Preparation

Nuclei were isolated from solid tissues using a Singulator 100 system (S2 Genomics) following the manufacturer's protocol. Debris was removed using a Percoll gradient. Nuclei concentration was determined using a LUNA‐FL Automated Fluorescence Cell Counter (Logos Biosystems), and morphology was examined by microscopy. The isolated nuclei were then incubated in a Transposition Mix containing Transposase.

Libraries were prepared using the Chromium controller (10x Genomics) according to the Chromium Single Cell Multiome ATAC + Gene Expression protocol (CG000338) to simultaneously generate paired single‐nucleus RNA sequencing (snRNA‐seq) and single‐nucleus ATAC sequencing (snATAC‐seq). Briefly, transposed nuclei were mixed with master mix and loaded with Single Cell Multiome gel beads and partitioning oil into a Chromium Chip J. The gel beads contained poly (dT) sequences for gene expression (GEX) library preparation and spacer sequences for ATAC library preparation.

Gel bead‐in‐emulsion incubation resulted in 10x barcoded DNA from transposed DNA (for ATAC) and 10x barcoded cDNA from polyadenylated mRNA (for GEX). After pre‐amplification, the PCR product was divided for separate ATAC and GEX library construction steps. For the ATAC library, P7 adapters and sample indices were added to pre‐amplified transposed DNA during library construction via PCR. For the GEX library, pre‐amplified cDNA underwent PCR amplification, followed by fragmentation, end repair, A‐tailing, adapter ligation, and index PCR.

### 4.11 Single‐Nucleus Multiome Library Quantification and Sequencing

The purified libraries were quantified using qPCR according to the qPCR Quantification Protocol Guide (KAPA Biosystems). Library quality was assessed using an Agilent Technologies 4200 TapeStation (Agilent Technologies). Sequencing was performed on a HiSeq platform (Illumina) following the read length in the user guide.

### 4.12 Pre‐Processing and Quality Control of Single‐Nucleus Multiome Data

Raw sequencing data (FASTQ files) were aligned to the *hg38* reference genome and quantified using Cell Ranger ARC v2.0.2. cellranger‐arc count pipeline produced barcoded count matrices for gene expression and ATAC data. These matrices and fragment files were then processed using Seurat v4.3.0 [60] and Signac v1.9.0 [61] software packages.

SoupX v1.6.2 [62] was employed to assess and mitigate background RNA contamination. The *autoEstCont* function was used to estimate contamination fractions, which were manually adjusted to 0.2 for tumor samples using *setContaminationFraction*. Background noise was subsequently eliminated using the *adjustCounts* function with default parameters. To generate a unified peak set from the snATAC‐seq data of all samples, we used the *reduce* function from GenomicRanges v1.46.1 [63]. These combined peaks were then quantified across all datasets. The scDblFinder v1.8.0 [64] was employed to identify and remove potential doublets. We implemented stringent quality control criteria, retaining cells with nCount_RNA > 1000, nCount_ATAC > 1000, and percent.mt < 3, and TSS.enrichment > 1.5. Sample‐specific thresholds were also implemented for each sample as follows:

HC1: nCount_ATAC < 20000 & nCount_RNA < 20000 & Nucleosome_Signal < 0.7

HC2: nCount_ATAC < 25000 & nCount_RNA < 20000 & Nucleosome_Signal < 0.7

Tumor1: nCount_ATAC < 20000 & nCount_RNA < 20000 & Nucleosome_Signal < 1.5

Tumor2: nCount_ATAC < 5000 & nCount_RNA < 15000 & Nucleosome_Signal < 1

### 4.13 Integrated Analysis Workflow of Single‐Nucleus Multiome Data

Post‐quality control, snRNA‐seq data were processed using the Seurat package. Normalization, variable feature identification (top 3000 genes), scaling, and PCA were performed. Batch effects were removed using Harmony v0.1.1 [65]. *RunUMAP* function was applied to the first 30 Harmony‐corrected PC dimensions for RNA data visualization.

The snATAC‐seq peak calling was conducted using MACS2 v2.2.6 [66] via the Signac package. Peaks overlapping with *hg38* blacklist regions were excluded. The resulting peak set was used to create a new chromatin assay within the Seurat object. Top features were identified (min.cutoff = 5), followed by TF‐IDF normalization and partial singular value decomposition. Batch effects of ATAC datasets were addressed using the *RunHarmony* function in the Harmony package on latent semantic indexing (LSI) components. UMAP visualization was generated via the *RunUMAP* function based on the second through 30th Harmony‐corrected LSI components.

WNN analysis was used to integrate gene expression and chromatin accessibility data. The *FindMultiModalNeighbors* function combined gene expression (PCA) and ATAC (LSI) data. WNN‐derived clusters were visualized using UMAP projection with *RunUMAP* function, setting nn.name to weighted.nn. Clustering was performed using the *FindClusters* function with a resolution of 0.8 and the SLM algorithm (algorithm 3), resulting in 28 clusters. Low‐quality cells exhibiting low gene detection with high mitochondrial reads and potential doublets showing double‐positive cell type marker gene expression were excluded from downstream analysis.

Cluster assignment relied on the concordance of both data modalities with established canonical lineage markers and functional subtype markers. This process ensured robust classification across major cell types [67, 68] and subsequent identification of epithelial [38, 69, 70, 71] and endothelial subtypes [72, 73].

### 4.14 Differential Expression and Chromatin Accessibility Analysis in Single‐Nucleus Multiome Data

Differential expression analysis was performed using Seurat v4.3.0. DEGs in major cell types were identified using the *FindAllMarkers* function (parameters: only.pos = TRUE, min.pct = 0.1, logfc.threshold = 1). Across malignant cell subpopulations, the logfc.threshold was adjusted to 0.58. DEGs between endothelial cell subclusters were determined using the *FindMarkers* function (min.pct = 0.1, logfc.threshold = 1). DARs between each cell type within tumor and HC tissues were identified using the *FindMarkers* function with min.pct = 0.02, test.use = LR, latent.vars = nCount_peaks, and logfc.threshold = 1. Genomic distribution of DARs was analyzed using HOMER v4.11.1 annotatePeaks.pl script with human genome *hg38* as reference. All significant DEGs and DARs were filtered with an adjusted *p*‐value < 0.05.

### 4.15 Functional Enrichment and Pathway Analysis of Single‐Nucleus Multiome Data

GO analysis of DEGs was performed using Metascape. Functional annotation of DARs was conducted using annotatePeaks.pl with *hg38* and ‐go options in HOMER software. The PROGENy package v1.16.0 [56] was employed to evaluate the activity of 14 key signaling pathways in single‐nucleus multiome data.

### 4.16 Gene‐Peak Linkage Analysis

Gene‐peak linkages were evaluated using the *LinkPeaks* function in Signac. Significantly linked peaks (*p*‐value < 0.05 and linkage score > 0) were integrated with DARs in epithelial cells, fibroblasts, and endothelial cells to find linked DARs with genes. Further overlapping with DEGs in each tumor‐enriched cell type compared with their normal compartments using *FindMarkers* function with min.pct = 0.1, logfc.threshold = 1, and adjusted *p*‐value below 0.05, were found DEGs‐linked DARs in each cell type.

### 4.17 Transcription Factor Motif Activity Analysis

TF binding motifs were annotated using the JASPAR 2020 database [74] and analyzed with chromVAR v1.16.0 [75] to estimate TF activity. These values were used to generate the motif heatmaps and feature plots. Differential motif activity was assessed using *FindAllMarkers* function with only.pos = TRUE, min.fxn = rowMeans, fc.name = avg_diff, logfc.threshold = 0.58, and adjusted *p*‐value < 0.05.

### 4.18 Copy Number Variation Analysis

CNV within epithelial cell subpopulations was defined using the inferCNV algorithm v1.10.1 (https://github.com/broadinstitute/infercnv). Raw count data, extracted from RNA assay in the Seurat object of epithelial cell clusters, were utilized to generate inferCNV object via *CreateInfercnvObject* function. Ciliated, mucous, and serous cells served as the reference group for this analysis. To detect CNV signals, we employed the *infercnv::run* function, setting the cutoff for the minimum average read count per gene to 0.1, as recommended for 10x Genomics data. Subsequently, the derived CNV data were integrated into the Seurat object using the *infercnv::add_to_seurat* function to facilitate visualization and downstream analyses.

### 4.19 Gene Signature Scoring

Module score for specific gene sets was calculated using the *AddModuleScore* function in Seurat. Signature gene lists were derived from the HALLMARK gene sets for hypoxia and glycolysis from the MSigDB, established HNSC transcriptional programs defined by Puram et al. [38], and angiogenesis signature genes from previous study [76]. Additionally, SNSCC‐associated signatures were generated using the top 100 upregulated and downregulated DEGs from bulk RNA‐seq analysis.

### 4.20 Gene Set Variation Analysis

Gene set variation analysis (GSVA) v1.42.0 [77] was used to generate signature scores for each TC subset (TC1‐TC5) in HNSC patients from TCGA data. To ensure compatibility with the TCGA gene expression data file, which uses the Ensembl Gene ID (ENSG) format, the DEGs defining each TC signature were converted to the ENSG format using the g:Profiler (http://biit.cs.ut.ee/gprofiler/). These TC subset signatures were constructed based on DEGs identified within the malignant epithelial cell compartment. The resulting GSVA scores were used for overall survival analysis of TC subpopulations. The complete gene lists comprising the TC signatures are provided in the Supporting Information.

### 4.21 Cell–Cell Communication Analysis

Intercellular signaling networks were analyzed using CellChat v1.6.1 [78]. We selected the secreted signaling pathways from the CellChat human database. The CellChatDB was supplemented with ADM‐RAMP2 and ADM‐RAMP3 ligand–receptor pairs to dissect adrenomedullin signaling pathways. CellChat objects were created using the *createCellChat* function. Ligand–receptor interactions between cell populations were determined using the CellChat pipeline with default parameters. For comparative analysis, independent CellChat objects were generated for SNSCC and HC tissues, then integrated using the *mergeCellChat* function. Differential interaction analysis was performed to identify significant changes in signaling pathways and ligand–receptor pairs between SNSCC and HC tissues. Communication networks were visualized using stacked bar, bubble, and circle plots. Significant interactions were defined using permutation testing with the default *p* < 0.05 threshold.

### 4.22 TCGA HNSC Cohort Analysis

Gene expression matrix and clinical data, including tumor stage, lymphovascular invasion status, and survival information of HNSC patients from TCGA, were obtained via the UCSC Xena web portal (https://xenabrowser.net/datapages/). We generated correlation analysis and overall survival curves, excluding patients with missing stage or survival period information. *ADM* gene expression and signature scores of each epithelial cell subpopulation were used to divide patients into two groups at the 50th percentile. *P* values were calculated using the log‐rank test.

### 4.23 Bulk Omni‐ATAC‐seq Library Preparation

RPMI 2650 cells, cultured under either hypoxic or normoxic conditions, were washed with Dulbecco's phosphate‐buffered saline (DPBS) (BIOWEST, Catalog no. L0615‐500) and harvested using TrypLE Express (Gibco, Catalog no. 12604013). Cells were pelleted by centrifugation at 100 × g for 7 min. For nuclei isolation, harvested cells were counted, and 60,000 cells were resuspended in 50 µL cold lysis buffer. The lysis buffer consisted of ATAC‐seq resuspension buffer (RSB) containing 10 mm Tris‐HCl pH 7.5 (Invitrogen, Catalog no. 15567‐027), 10 mm NaCl (Invitrogen, Catalog no. AM9759), and 3 mm MgCl_2_ (Invitrogen, Catalog no. AM9530G) supplemented with 0.1% NP40 (Roche, Catalog no. 11332473001), 0.1% Tween‐20 (Roche, Catalog no. 11332465001), and 0.01% digitonin (Promega, Catalog no. G9441). This cell lysis reaction was incubated on ice for 3 min, followed by the addition of 1 mL of RSB with 0.1% Tween‐20 (without NP40 or digitonin) for nuclei wash. Nuclei were then centrifuged for 10 min at 500 × g at 4 °C. The supernatant was removed, and isolated nuclei were resuspended in 50 µL of transposition mix (25 µL TD Tagment DNA Buffer (Illumina, Catalog no. 15027866), 2.5 µL TDE1 Tagment DNA Enzyme (Illumina, Catalog no. 15027865), 16.5 µL DPBS, 0.5 µL 1% digitonin, 0.5 µL 10% Tween‐20, and 5 µL nuclease‐free water). Transposition reactions were incubated at 37 °C for 30 min in a thermomixer with shaking at 1000 rpm. Transposed DNA was cleaned up using the MinElute Reaction Cleanup Kit (Qiagen, Catalog no. 28206) and subsequently pre‐amplified for 5 cycles using NEBNext High‐Fidelity 2X PCR Master Mix (NEB, Catalog no. M0541) with Illumina/Nextera adapter primers. The amplification profile was manually assessed to determine the number of additional cycles needed for optimal amplification, as previously described [79]. Final amplified libraries were purified using AMPure XP beads (Beckman Coulter, Catalog no. A63880) and stored at −80 °C. Library quality was validated using the Agilent High Sensitivity D1000 kit (Agilent, Catalog no. 5067‐5584, 5067‐5585) before high‐throughput sequencing on an NovaSeq 6000 Sequencer.

### 4.24 Bulk Omni‐ATAC‐seq Analysis

Raw ATAC‐seq reads were trimmed using Trim Galore v0.6.4 with default parameters to ensure data quality. The trimmed reads were then aligned to the human reference genome (*hg38*) using Bowtie2 v2.3.4.1 [80] with the following parameters: –*very‐sensitive –no‐discordant ‐X 2000*. To identify open chromatin regions (peaks) over background signals, HOMER software v4.11.1 was utilized, employing the makeTagDirectory and findPeaks commands with parameters optimized for ATAC‐seq data (*‐style factor ‐o auto ‐minDist 100 ‐size 60*). For downstream visualization of signal tracks, BigWig files were generated using the bamCoverage command from deepTools v3.4.3 [81], with the parameters: –*binSize 10 –normalizeUsing RPGC –effectiveGenomeSize 2913022398*. This RPGC‐based normalization used an *hg38* effective genome size of 2,913,022,398 bp, enabling accurate visual comparison across samples.

### 4.25 ELISA

CM was prepared from RPMI 2650 cells. Cells were seeded in 12‐well plates at a density of 5 × 10^5^ cells/well in RPMI 1640 medium containing 10% FBS and 1% penicillin/streptomycin. After 48 h, cells were washed with PBS, and the growth medium was replaced with fresh, serum‐free RPMI 1640. CM was then collected after an additional 24 h of incubation under either normoxic or hypoxic conditions. Hypoxic conditions were established by placing cells in a hypoxia chamber, maintained at 37 °C in an atmosphere of 1% O_2_ and 5% CO_2_. Collected supernatants were clarified by centrifugation at 1000 × g for 5 min to remove cellular debris, followed by filtration through a 0.22 µm filter (Corning, Catalog no. 431153). VEGF protein levels in CM collected from RPMI 2650 cells (normoxia and hypoxia) were quantified using a human VEGF ELISA kit (Invitrogen, Catalog no. KHG0111) according to the manufacturer's instructions. Absolute concentrations were determined by interpolation from a standard curve generated with known concentrations of recombinant human VEGF provided in the kit.

### 4.26 Flow Cytometry

CM was prepared from RPMI 2650 cells using the procedure described above (refer to ELISA section), but CM was collected after an additional 48 h of incubation under hypoxic conditions. HUVECs were seeded into a six‐well plate at a density of 2.5 × 10^5^ cells/well. Prior to cell seeding, the plates were coated with 0.1% gelatin (Sigma–Aldrich, Catalog no. G1890) and allowed to solidify for 1 h at 37°C. Cells were then cultured overnight and subsequently serum‐starved by incubating in Medium 200 containing 2% FBS for an additional 24 h. Following serum starvation, cells were treated with 100 nM ADM (Phoenix Pharmaceuticals, Catalog no. 010‐01) or hypoxic tumor CM for 15 min. For VEGF neutralization, the CM was pre‐incubated with 10 µg/mL Bevacizumab (MedChemExpress, Catalog no. HY‐P9906) for 1 h prior to treatment. Immediately following treatment, cells were harvested using TrypLE Express (Gibco, Catalog no. 12604013). Cells were fixed using pre‐warmed (37 °C) fixation buffer (BioLegend, Catalog no. 420801) at 37 °C for 15 min. Following washing, permeabilization was carried out using pre‐chilled (−20 °C) True‐Phos Perm Buffer (BioLegend, Catalog no. 425401) for 60 min at −20 °C. Cells were subsequently labeled separately with antibodies targeting Phospho‐ERK1/2 (Thr202/Tyr204) (BioLegend, Catalog no. 369510) and Phospho‐CREB1 (Ser133) (BioLegend, Catalog no. 632556) for 30 min at room temperature. Stained samples were analyzed using a CytoFLEX (Beckman Coulter) and processed with FlowJo 11.0.2. The experiment was performed in triplicate.

### 4.27 Immunofluorescence Staining

IF staining was performed on SNSCC patient tumor tissues. Specimens were fixed in 4% paraformaldehyde in PBS and then paraffin‐embedded using standard histological procedures. Sections, 4 µm thick, were prepared. Heat‐induced antigen retrieval was accomplished by boiling sections in 10 mm citrate buffer (pH 6.0), followed by cooling to room temperature for 20 min. After antigen retrieval, specimens were incubated overnight at 4 °C with primary antibodies: anti‐GLUT1 (Abcam, Catalog no. ab115730, 1:100) and anti‐DLL4 (Cell Signaling Technology, Catalog no. 96406S, 1:100). Secondary antibody incubation was performed for 2 h at room temperature using donkey anti‐rabbit Alexa Fluor 555 (Invitrogen, Catalog no. A‐31572, 1:200). Nuclear counterstaining was achieved with 4’,6‐diamidino‐2‐phenylindole (DAPI) (Invitrogen, Catalog no. D1306, 1:1,000). Stained sections were then imaged using a confocal laser scanning microscope (LSM 900, Carl Zeiss). IF images were analyzed for quantitative spatial assessment using ImageJ software v.1.54 g. Specifically, the percentage of positive staining area per field of view was calculated using marker‐specific fixed threshold parameters to ensure consistent quantification across samples.

### 4.28 Statistical Analysis

Statistical analyses were conducted using R software (versions 4.1.0 and 4.1.1) and GraphPad Prism 10. Results are presented as mean ± standard error of the mean (SEM) unless otherwise specified. Statistical significance was defined as *p*‐value < 0.05, as determined by two‐tailed unpaired *t*‐test, two‐tailed paired *t*‐test, or one‐way ANOVA where appropriate. In box plots, center lines show medians, box limits indicate 25th and 75th percentiles, and whiskers extend to minimum and maximum values or 10th and 90th percentiles, as specified in figure legends. Correlations between variables were assessed using Pearson's correlation coefficient. Statistical significance of correlations was evaluated using two‐tailed tests, with *p*‐value < 0.05 considered significant. Survival analyses were performed using the Kaplan–Meier method. Differences in survival curves were assessed using the log‐rank test. Patients were stratified into high and low groups based on the median expression level of genes of interest or signature scores.

### 4.29 Code Availability

All computational analyses performed for this study, including multi‐modal data processing and figure generation, are based on publicly available software packages as detailed in the Methods section. The complete computational pipeline, including all R scripts and analytical procedures necessary for reproducing the single‐nucleus multiome data analysis and figure generation, is publicly available in the GitHub repository: https://github.com/kangk1204/SNSCC_HC_multiome.

### 4.30 Data Availability

The datasets generated in this study are available in the GEO repository under SuperSeries accession GSE278149. This SuperSeries is composed of the following SubSeries: GSE278145 (bulk RNA‐seq), GSE278138 (DNA methylation EPIC array), GSE277797 (snRNA/ATAC‐seq), GSE298563 (RPMI 2650 bulk RNA‐seq), and GSE298564 (RPMI 2650 bulk ATAC‐seq). The public dataset analyzed in this study is available under the GEO accession GSE189778 [19]. The HNSC dataset from TCGA was obtained from the UCSC Xena web portal (https://xenabrowser.net/datapages/).

## Author Contributions

C.Y. conceptualized, designed, and performed most of the experiments and conducted bioinformatic analyses. Jaewoo P., J.Y.J., J.N., J.L., G.K., M.S.Y., Y.S.C., and S.L. contributed to the experiments. Keunsoo K. and Jihwan P. contributed to bioinformatic analyses and provided expertise. Kyuho K. and J.H.K. conceptualized and supervised the study and edited the manuscript. All authors reviewed and provided input on the manuscript.

## Conflicts of Interest

The authors declare no conflicts of interest.

## Supporting information




**Supporting File 1**: advs73625‐sup‐0001‐Figures.docx.

**Supporting File 2**: advs73625‐sup‐0002‐Table.xlsx.

## Data Availability

The data that support the findings of this study are available from the corresponding author upon reasonable request.
