# Supplementary material for: Single‐Nucleus Multi‐Omics Reveals Hypoxia‐Driven Angiogenic Programs and Their Epigenetic Control in Sinonasal Squamous Cell Carcinoma
Source: Adv Sci (Weinh). 2026 Jan 7;13(12):e10302. doi: 10.1002/advs.202510302 (PMC12948189; doi:10.1002/advs.202510302)
Supplement: Supplementary file 1 — Supporting File 1: advs73625‐sup‐0001‐Figures.docx. [file ADVS-13-e10302-s001.docx]

**Supporting information**

**Single-Nucleus Multi-omics Reveals Hypoxia-Driven Angiogenic Programs and Their Epigenetic Control in Sinonasal Squamous Cell Carcinoma**

Chaelin You^1^, Jaewoo Park^1^, Jung Yeon Jang^2^, Junho Noh^1^, Jaehyun Lee^1^, Geunho Kwon^1^, Myeong Sang Yu^2^, Yoo-Sam Chung^2^, Seung-Jun Lee^3^, Keunsoo Kang^4^, Jihwan Park^5^, Ji Heui Kim^2,*^, Kyuho Kang^1,*^

^1^Department of Biological Sciences and Biotechnology, Chungbuk National University, Cheongju 28644, Korea

^2^Department of Otorhinolaryngology-Head and Neck Surgery, Asan Medical Center, University of Ulsan College of Medicine, 88 Olympic-ro 43-gil, Songpa-gu, 05505, Seoul, Republic of Korea

^3^Research Institute of Pharmaceutical Sciences and College of Pharmacy, Seoul National University, Seoul, Korea

^4^Department of Microbiology, College of Science & Technology, Dankook University, Cheonan 31116, Korea

^5^School of Life Sciences, Gwangju Institute of Science and Technology (GIST), Gwangju, Republic of Korea

^*^**Corresponding author**: Kyuho Kang, [kangk@cbnu.ac.kr](mailto:kangk@cbnu.ac.kr); Ji Heui Kim, [jhkim0217@amc.seoul.kr](mailto:jhkim0217@amc.seoul.kr)

**This PDF file includes:**

Figures S1 to S7

**Figure S1. Transcriptional profiling and cellular composition analysis of SNSCC.** (*A*) Principal component analysis (PCA) of bulk RNA sequencing (RNA-seq) data comparing sinonasal squamous cell carcinoma (SNSCC) tumor and normal tissue samples. (*B*) Expression analysis of selected genes associated with Gene Ontology (GO) terms identified in Figure 1*C* from bulk RNA-seq. Data represent mean ± SEM; statistical significance determined by two-tailed unpaired t-test (**p* < 0.05, ***p* < 0.01, ****p* < 0.001, *****p* < 0.0001). (*C*) Top six enriched hallmark pathways from Gene Set Enrichment Analysis (GSEA) comparing SNSCC tumor to normal samples. (*D*) Uniform manifold approximation and projection (UMAP) visualization of 18,319 cells clustered by weighted nearest neighbor (WNN) analysis, revealing 28 distinct cell populations. (*E*) Expression patterns of established cell type markers across 28 identified clusters; corresponding cell type annotations are indicated. (*F*) Distribution of quality control metrics across cell clusters: number of detected genes (nFeature_RNA), mitochondrial gene percentage (percent.mt), and scDblFinder-calculated doublet scores. (*G*) Differential abundance analysis of major cell types between SNSCC and healthy control (HC) tissues; significance threshold was set at false discovery rate (FDR) < 0.05 and |log2 fold change| > 1. (*H*) CIBERSORTx deconvolution analysis of 22 immune cell populations in SNSCC and normal tissues (100 permutations; mean values shown). (*I*) Comparative analysis of macrophage (M0 + M1 + M2) and plasma cell scores between SNSCC and normal tissues. Data represent mean ± SEM; statistical significance determined by two-tailed unpaired t-test (**p* < 0.05, ***p* < 0.01, ****p* < 0.001, *****p* < 0.0001). (*J*) Expression analysis of *CD68* and *JCHAIN* comparing SNSCC to normal samples from bulk RNA-seq. Data represent mean ± SEM; statistical significance determined by two-tailed unpaired t-test (**p* < 0.05, ***p* < 0.01, ****p* < 0.001, *****p* < 0.0001).

**Figure S2. Gene Ontology analysis reveals distinct functional signatures across cell types in SNSCC and healthy tissue.** (*A*) Statistical significance of GO terms enriched in cell type-specific marker genes within SNSCC tumor tissues. (*B*) Statistical significance of GO terms enriched in cell type-specific marker genes within HC tissues. (*C*) Expression patterns of selected genes associated with representative GO terms shown in (*B*), in epithelial cells (Left) and fibroblasts (Right) within HC tissues. (*D*) Feature plots showing the expression distributions of representative genes (*KRT6A*, *NDRG1*, *FAP*, *ANGPT2*, *ADAM8*, and *IL2RB*) identified in Figure 2*B* across all cell populations in SNSCC and HC tissues.

**Figure S3. Cell type-specific analysis of chromatin accessibility and transcription factor activity in SNSCC.** (*A*) Quantitative analysis of differentially accessible regions (DARs) in SNSCC versus HCs, showing total number (Upper) and log2 fold change (Middle) for epithelial cells, fibroblasts, and endothelial cells, with comparative DAR counts in immune populations (Lower). (*B*) Distribution of total DARs and differentially expressed gene (DEG)-linked DARs across epithelial, fibroblast, and endothelial populations in SNSCC and HC samples. (*C*) HOMER-based genomic annotation of DEG-linked DARs in epithelial, fibroblast, and endothelial populations from SNSCC and HC samples. (*D*) GO analysis of upregulated DEG-linked DARs identified in Figure 3*A* for epithelial cells (Upper), fibroblasts (Middle), and endothelial cells (Lower), performed using HOMER. (*E*) Expression distribution of selected genes associated with opened DAR-linked upregulated DEGs identified in Figure 3*A* for epithelial cells (Upper), fibroblasts (Middle), and endothelial cells (Lower) in SNSCC. (*F*) ChromVAR motif activity scores for the top 10 enriched transcription factor (TF) binding motifs across cell types in SNSCC and HC samples. (*G*) Comparative analysis of *FOSL2* and *JUNB* expression between SNSCC and normal samples from bulk RNA-seq. Data represent mean ± SEM; statistical significance determined by two-tailed unpaired t-test (**p* < 0.05, ***p* < 0.01, ****p* < 0.001, *****p* < 0.0001).

**Figure S4. Genome-wide DNA methylation analysis reveals altered transcription factor networks in SNSCC.** (*A*) Multidimensional scaling analysis (MDS) of EPIC array methylation profiles from SNSCC and normal tissues, incorporating data from the current study and GSE189778. (*B*) Quantitative analysis of methylation levels (β-values) at hypo- and hypermethylated differentially methylated positions (DMPs) in SNSCC versus normal samples. Box plots show 25th to 75th percentiles with whiskers at 10th and 90th percentiles; center lines indicate medians. Statistical significance determined by two-tailed unpaired t-test. (*C*) *De novo* motif analysis identifying TF binding motifs enriched in hypomethylated DMPs within promoter-proximal regions. (*D*) Comparative analysis of *NFIB* and *NFIX* expression between SNSCC and normal samples from bulk RNA-seq. Data represent mean ± SEM; statistical significance determined by two-tailed unpaired t-test (**p* < 0.05, ***p* < 0.01, ****p* < 0.001, *****p* < 0.0001). (*E*) UMAP visualization of *NFIB* and *NFIX* expression patterns (Upper) and corresponding motif activity scores (Lower), stratified by tissue type. (*F*) Overlap analysis identifying hypermethylated genes showing concurrent transcriptional downregulation (hyper-down) in SNSCC compared to normal tissue. (*G*) Enrichment analysis of TF binding motifs in hypomethylated upregulated DMPs within promoter-proximal regions of SNSCC, analyzed using HOMER. Circle size indicates statistical significance; color coding represents TF families.

**Figure S5. Single-cell analysis reveals distinct malignant epithelial subpopulations with prognostic significance in SNSCC.** (*A*) UMAP visualization of epithelial populations in SNSCC and HC samples based on WNN analysis, colored by sample origin. (*B*) UMAP projection revealing 15 distinct epithelial cell clusters. (*C*) Expression patterns of cell type-specific marker genes across identified epithelial clusters. (*D*) Quantitative distribution of epithelial populations between SNSCC and HC tissues. (*E*) Distribution of signature scores derived from top 100 upregulated (Upper) and downregulated (Lower) DEGs from bulk RNA-seq across epithelial populations. (*F*) Heatmap of DEGs across malignant subpopulations (adjusted *p* < 0.05, log2 fold change > 0.58). (*G*) Statistical significance of GO terms enriched among DEGs across malignant cell subtypes. (*H*) Intersection analysis of DEGs across malignant cell subtypes and upregulated genes identified in bulk RNA-seq comparison of SNSCC versus normal tissue. (*I*) Comparative analysis of TC3, TC4, and TC5 signature scores between SNSCC and normal samples from bulk RNA-seq. Data represent mean ± SEM; statistical significance determined by two-tailed unpaired t-test (**p* < 0.05, ***p* < 0.01, ****p* < 0.001, *****p* < 0.0001; ns, not significant). (*J*) Dot plot showing the enrichment of head and neck squamous cell carcinoma (HNSC) signatures defined by Puram et al. across tumor cell clusters. Dot size indicates the percentage of expressing cells; color represents average expression. (*K*) Kaplan-Meier analysis of overall survival in The Cancer Genome Atlas (TCGA) HNSC cohort stratified by TC3 (Left), TC4 (Middle), and TC5 (Right) signature scores (50th percentile cutoff); statistical significance determined by log-rank test. TC3 signature (HR = 1.02; 95% CI = 0.78–1.33); TC4 signature (HR = 1.02; 95% CI = 0.78-1.33); TC5 signature (HR = 1.28; 95% CI = 0.98-1.67).

**Figure S6. Single-cell mapping defines endothelial subtypes and their pro-angiogenic communication with hypoxic tumor cells in SNSCC.** (*A*) Comparative analysis of significantly upregulated signaling pathway activities in SNSCC versus normal tissues, quantified using PROGENy. (*B*) UMAP visualization and quantitative distribution of hypoxia (Left) and glycolysis (Right) signature scores across epithelial populations. (*C*) Comparative analysis of enriched signaling pathways mediating epithelial-to-endothelial cell communication in SNSCC and HC samples. Statistically significant pathway enrichments (*p* < 0.05) across disease conditions are indicated in bold. (*D*) Circos visualization of CALCR, MIF, VEGF, and ncWNT signaling networks among cell populations; edge thickness indicates interaction probability. (*E*) UMAP visualization of endothelial populations in SNSCC and HC samples based on WNN analysis, revealing seven distinct clusters prior to doublet removal. (*F*) Distribution of scDblFinder-calculated doublet scores across endothelial subclusters. (*G*) Differential gene expression analysis comparing EC2 to other endothelial subclusters. Significant DEGs defined by |log2 fold change| > 1 and adjusted *p* < 0.05. Nonsignificant genes are shown in gray. (*H*) GO terms enriched in upregulated genes from the EC2 population identified in (*G*). (*I*) Expression patterns of selected upregulated genes in EC1 and EC2 populations relative to other endothelial subclusters identified in Figure 6*F* and S6*G*. (*J*) Circos visualization of predicted ligand-receptor interactions among cell populations: ADM-CALCRL, ADM-RAMP2, and VEGFA-VEGFR2; edge thickness indicates interaction probability. (*K*) Quantification of immunofluorescence (IF) staining assessing the tumor area occupied by key cell populations in SNSCC patient tissues. The graph displays the percentage area of GLUT1 expression and DLL4 expression. Individual dots represent measurements from individual patient samples (*n* = 3). Data represent mean ± SEM.

**Figure S7. Multi-omics analysis delineates chromatin and transcriptional regulation of pro-angiogenic factors with clinical relevance in SNSCC.** (*A*) PCA of bulk RNA-seq data comparing RPMI 2650 cells under hypoxic and normoxic conditions. (*B*) PCA of bulk ATAC-seq data comparing RPMI 2650 cells under hypoxic and normoxic conditions. (*C*) ATAC-seq tracks displaying enhanced signal at the *ENO1* and *HK2* loci in RPMI 2650 cells under hypoxic (red) compared to normoxic (green) conditions. The highlighted regions (orange boxes) correspond to the specific open chromatin regions (OCRs) shown in (*D*). (*D*) Quantification of ATAC-seq tag counts at specific OCRs within the *ENO1* and *HK2* loci in RPMI 2650 cells under hypoxic and normoxic conditions. Values represent normalized tag counts (mean ± SEM from *n* = 3 technical replicates); statistical significance determined by two-tailed unpaired t-test (**p* < 0.05, ***p* < 0.01, ****p* < 0.001, *****p* < 0.0001). (*E*) Validation of gene expression for *MIF* and *HK2* in RPMI 2650 cells under normoxic or hypoxic conditions, with or without treatment with SB203580. Data represent mean ± SEM; statistical significance determined by one-way ANOVA (**p* < 0.05, ***p* < 0.01, ****p* < 0.001, *****p* < 0.0001). (*F*) Validation of gene expression for *MIF* and *HK2* in RPMI 2650 cells under normoxic or hypoxic conditions, with or without treatment with PX-478. Data represent mean ± SEM; statistical significance determined by one-way ANOVA (**p* < 0.05, ***p* < 0.01, ****p* < 0.001, *****p* < 0.0001). (*G*) Functional *in vitro* signaling analysis assessing ERK phosphorylation levels in human umbilical vein endothelial cells (HUVECs) treated with ADM. Data represent mean ± SEM; statistical significance determined by two-tailed unpaired t-test (**p* < 0.05, ***p* < 0.01, ****p* < 0.001, *****p* < 0.0001). (*H*) RT-qPCR analysis quantifying *ADM*, *MIF*, and *VEGFA* transcript levels in SNSCC and paired normal tissues. Data represent mean ± SEM (*n* = 7); statistical significance determined by two-tailed unpaired t-test (**p* < 0.05, ***p* < 0.01, ****p* < 0.001, *****p* < 0.0001). (*I*) Comparative analysis of *ADM*, *MIF*, and *VEGFA* expression between HNSC and normal tissues from TCGA dataset. Data represent mean ± SEM (*n* = 501 for HNSC, *n* = 44 for normal tissues); statistical significance determined by two-tailed unpaired t-test (**p* < 0.05, ***p* < 0.01, ****p* < 0.001, *****p* < 0.0001). (*J*) Genome browser view of DNA methylation at the *ADM* locus in SNSCC (*n* = 3, red) and normal tissues (*n* = 3, blue). Tracks represent average beta-values at individual CpG sites across samples in each group. (*K*) Integrated visualization of ATAC-seq signal intensity and gene expression levels for *ADM* in epithelial cells from SNSCC and HC samples; peak coordinates and peak-gene associations are indicated. (*L*) *ADM* expression in normal tissue and HNSC samples from the TCGA dataset, stratified by tumor stage (Left; Normal: *n* = 44, Stage I-II: *n* = 119, Stage III-IV: *n* = 374), lymphovascular invasion status (Middle; No: *n* = 218, Yes: *n* = 122), and survival status (Right; Alive: *n* = 282, Dead: *n* = 219). Data represent mean ± SEM. Statistical significance for comparisons among normal tissue and tumor stages was determined by one-way ANOVA, and for lymphovascular invasion and survival status by two-tailed unpaired t-test (**p* < 0.05, ***p* < 0.01, ****p* < 0.001, *****p* < 0.0001). (*M*) Correlation analysis between *ADM* expression and *VEGFA* expression in TCGA-HNSC dataset (*n* = 501). Pearson correlation was used to assess the association. Correlation coefficient (R) and *p*-values are indicated on the plots. (*N*) Kaplan-Meier analysis of overall survival in TCGA HNSC cohort stratified by *ADM* expression (50th percentile cutoff); statistical significance determined by log-rank test (HR = 1.37; 95% CI = 1.05-1.78).
